# A novel oyster shell biocomposite for the efficient adsorptive removal of cadmium and lead from aqueous solution: Synthesis, process optimization, modelling and mechanism studies

**DOI:** 10.1371/journal.pone.0294286

**Published:** 2024-02-22

**Authors:** Abdulrahman Oyekanmi Adeleke, C. O. Royahu, Akil Ahmad, Temitope T. Dele-Afolabi, Mohammed B. Alshammari, Monzur Imteaz

**Affiliations:** 1 Institute of Energy Infrastructure (IEI), Universiti Tenaga Nasional (UNITEN), Putrajaya Campus, Jalan IKRAM-UNITEN, Kajang, Selangor, Malaysia; 2 Chemistry Department, College of Sciences and Humanities, Prince Sattam bin Abdulaziz University, Al-Kharj, Saudi Arabia; 3 Institute of Power Engineering (IPE), Universiti Tenaga Nasional (UNITEN), Putrajaya Campus, Jalan IKRAM-UNITEN, Kajang, Selangor, Malaysia; 4 Department of Civil and Construction Engineering, Centre for Sustainable Infrastructure and Digital Construction, Swinburne University of Technology, Melbourne, Australia; Qatar University, QATAR

## Abstract

This study highlights the effectiveness of oyster shell biocomposite for the biosorption of Cd(II) and Pb(II) ions from an aqueous solution. The aim of this work was to modify a novel biocomposite derived from oyster shell for the adsorption of Cd(II) and Pb(II) ions from aqueous solution. The studied revealed the specific surface BET surface area was 9.1476 m^2^/g. The elemental dispersive x-ray analysis (EDS) indicated that C, O, Ag, Ca were the predominant elements on the surface of the biocomposite after which metals ions of Cd and Pb were noticed after adsorption. The Fourier transform Irradiation (FT-IR) revealed the presence of carboxyl and hydroxyl groups on the surface. The effect of process variables on the adsorption capacity of the modified biocomposite was examined using the central composite design (CCD) of the response surface methodology (RSM). The process variables which include pH, adsorbent dose, the initial concentration and temperature were the most effective parameters influencing the uptake capacity. The optimal process conditions of these parameters were found to be pH, 5.57, adsorbent dose, 2.53 g/L, initial concentration, 46.76 mg/L and temperature 28.48°C for the biosorption of Cd(II) and Pb(II) ions from aqueous solution at a desirability coefficient of 1. The analysis of variance (ANOVA) revealed a high coefficient of determination (R^2^ > 0.91) and low probability coefficients for the responses (P < 0.05) which indicated the validity and aptness of the model for the biosorption of the metal ions. Experimental isotherm data fitted better to the Langmuir model and the kinetic data fitted better to the pseudo-second-order model. Maximun Cd(II) and Pb(II) adsorption capacities of the oyster shell biocomposite were 97.54 and 78.99 mg/g respectively and was obtained at pH 5.56 and 28.48°C. This investigation has provided the possibility of the utilization of alternative biocomposite as a sustainable approach for the biosorption of heavy metal ions from the wastewater stream.

## 1. Introduction

The discharge of wastes generated from the industry on the environment and the receiving water is a persistent environmental issue [1]. In recent years, rapid industrial expansion has increased the discharge of heavy metals into the environment and the receiving water [2]. The bioaccumulation of heavy metals and their non-degradable nature has affected the water system and the aquatic biota. Heavy metal is toxic and hazardous to human health even at low concentrations [3]. Heavy metals of Cd and Pb are the most common pollutants generated from industry and they constitute detrimental effects causing different forms of health disorders and diseases [1]. Therefore, the removal of these metals before discharge is essential for the preservation of water quality and the ecosystem.

Several treatment processes for the removal of Cd(II) and Pb(II) ions has been explored among which adsorption has a comparative advantage due to the simplicity of its design and operation, its ease of access to precursor and its cost-effective treatment techniques with guaranteed high efficiency. The adsorption abilities of several low-cost adsorbents for heavy metals adsorption has been reported, such as the use of coconut shells, corn cobs, zeolite, fly ash, durian peels, empty fruit bunches, and maize cobs [4–9]. Recently, the utilization of calcium carbonate (CaCO_3_) containing adsorbents has attracted widespread interest because it is a very inexpensive material that is commonly found in nature, they are non-toxic and are also environmentally friendly [10]. The oyster shells, which is hugely deposited wastes across the coastal areas are very rich in CaCO_3_[11]. However, the recycling of these wastes is favoured over its non-utilization being a potential source of noxious odours generated from gases such as NH_3_, H_2_S, and amines as a consequence of the decaying remnant of flesh attached to the shells [12]. The degradation of oyster shells has the potential to increase microbial activities which may constitute other forms of environmental issues if not properly managed [13]. Therefore, using oyster wastes for pollutant remediation is significant to ensure environmental protection. CaCO_3_ containing adsorbents has been used for heavy metals removal such as Cu^2+^, Hg^2+^, Cr^3+^, Mn^2+^, Zn^2+^, Cd^2+^, and Pb^2+^ [14–17]. The improvement of the adsorptive capacity of CaCO_3_ enriched adsorbents for the removal of heavy metals has become a major research focus in the field of wastewater engineering and pollutant remediation.

Fan et al. [18] modified nano-iron/oyster shell composites via in situ modification method for the adsorption of heavy metals from an aqueous solution. The authors revealed 100% removal capacity of the adsorbent for the removal of As(II) and As(III), which was achieved at pH of 6.8. Oyster shells has been utilized as a carrier to load humic acid-modified Fe_3_O_4_ nanoparticles via the co-precipitation method [19]. It was revealed that the maximum adsorption capacity was found to be 141.57 mg·g^−1.^ Wu et al. [20] has reported ion-imprinted oyster shell material for targeted removal of Cd(II) from aqueous solution. However, the interaction of process parameters towards enhanced removal capacity of the adsorbent was not addressed in the study. According to literature, oyster shell-based adsorbent has been utilized for heavy metals removal. Table 1 presents the adsorption efficiency of oyster shell-based adsorbents in the literature.

**Table 1:** Oyster shell based adsorbent and removal efficiency for heavy metal removal.

Adsorbent	Heavy metals remediated	Qe (mg/g), Efficiency (%)	Reference
Vaterite granules oyster shell	Pb^2+^, Cr^3+^, Fe^3+^, Cu^2+,^	Pb^2+^ (99.9%), Cr^3+^ (99.5%), Fe^3+^ (99.3%), Cu^2+^ (57.1%)	[21]
PVA/waste oyster shell powder composite	Cu^2+^, Cd^2+^	Cu^2+^ 26.7911, Cd^2+^ 18.1439	[22]
Calcium silicate hydrates derived oyster shell	Cu^2+^, Cr^2+^	Cu^2+^ 203.25, 256.41	[23]
Oyster shell bricks	Zn(II), Ni(II) and Mn(II)	Zn (II) 81.43%, Ni(II) 86.91% and 71.26%	[24]
Layered composite metal hydroxide(LDHs) based oystershell	Phosphate	26.41	[25]
OysterShell/Fe3O4 Nanoparticles/Humic Acid Composite	Hg^2+^	141.57	[26]
Chitosan derived from oyster (Anadara inflata) shell	Cu^2+^	70%	[27]
Oyster shell powder–treated rice husk ash composite	As(III)	26.20	[28]

Moreso, the synergistic interaction of process variables towards enhanced removal capacity of oyster shell-based adsorbent for the adsorption of Cd and Pb has not been reported in the literature which this study intends to address using response surface methodology (RSM) optimization. The use of central composite design (CCD) is preferable to design of experiment and optimization compared to the use of Box-Behnken design (BBD). This is because CCD allows for extreme factor combinations of operational parameters, whereas BBD does not examine borderline regions [29]. The synergistic effect of the combination of operational process variables is important for the assessment of the efficiency of the response parameters.

The application of oyster shell as a suitable adsorbent for metal removal is determined by its morphological characteristics that enables the binding of metal ions to the surface of the adsorbent. The utilization of oyster shells in different forms such as the preparation of vaterite calcium carbonate granules from discarded oyster shells has been reported [21], the use of oyster shell powder did not consider the synergistic interactions of operational parameters [30]. which is the focus of our study. In this work, discarded oyster shells were modified as novel biocomposite for the adsorption of Cd(II) and Pb(II) ions from solution. The effect of the process variables were examined using central composite design (CCD) of the response surface methodology (RSM) Furthermore, experimental data and characterization results were used to describe the mechanisms of Cd(II) and Pb(II) adsorption. To the best of our knowledge, no research has addressed the removal of Cd(II) and Pb(II) from aqueous solutions via RSM-CCD optimization using oyster shell biocomposite. Our study aims to fill this gap by the optimization of adsorption efficiency of the adsorbent via pH, initial dye concentration, adsorbent dosage, and temperature. Under optimized process condition, batch isotherm and kinetic studies were conducted including thermodynamic parameters of adsorption.

This study offers new perspectives for heavy metals removal and also aims to reveal the application potentials of the oyster shell biocomposite for pollution control.

## Materials and methods

### 2.1 Adsorbent

The oyster shells were collected at Pulau Bidan Island in Kedah North of Malaysia without the need for permit from local legislation and institution. The discarded precursor was thoroughly washed with deionized water and were oven dried at 110°C before calcination for the removal of surface impurities. The powdered adsorbent was obtained after crushing and sieving using a 75-μm sieve size. 20 g of oyster shell powder was taken and was pre-treated by boiling in demineralized water (DMW) for 1 h. The pre-treated oyster shell powder was oven-dried at 80°C for 24 h and was calcined at 800°C for 2 h in a muffle furnace to obtain a fine calcined powder. 10 g of calcined powder was dispersed in 100 ml (2M) H_2_SO_4_, and 100 ml (2M) HNO_3_ was added to the mixture. The solution was agitated for 2 h using the magnetic stirrer and was left for 24 h to improve the surface properties of the adsorbent material. After which sample was oven-dried at 80°C for 24 h and was stored in a closed vessel.

To modify the oyster shell biocomposites, firstly, 5 g of oyster shell powder was put into a conical flask and 100 mL demineralized water DMW was added. The resultant solution was later ultrasonicated for 30 min after which 100 mL of silver nitrate solution (0.1M) was mixed and the supernatant was sonicated for 15 min [31]. After then, 20 mL of lemon juice which served as a reducing agent was slowly added into the mixture. The pH of the mixture was adjusted using 0.1M HCl and 0.1M NaOH until solution pH 8 was achieved, this was followed by vigorous stirring of the mixture for 12 h. After then, the analyte was filtered and was oven dried for 24 h at 80°C to obtain a precipitate. The oyster shell biocomposite was stored in a tightly packed glass vial. Samples were taken for characterization and for batch adsorption experiments for the removal of Cd(II) and Pb(II) from aqueous solution.

### 2.2 Characterization

The morphology of the modified oyster shell biocomposite adsorbent was conducted before and after adsorption using scanning electron microscopy (SEM). Prior to the morphological analysis, the samples were dissolved in alcohol, drop cast on a (1 cm × 1 cm) silicon substrate, and were then gold plated for 50 s. The morphology of the sample was observed using a JEOL JSM-6380 microscope which was obtained under acceleration voltage of 0.5–30 kV. Energy dispersive X-ray spectroscopy (EDS) was obtained at a voltage of 20.00 kV in a high vacuum. The surface functional properties of the investigated samples were detected before and after adsorption using Fourier transform infrared spectroscopy (FT-IR) (Vertex70, Bruker, Germany in the range of 400–4000 cm^−1^. To identify the main functional groups, the Fourier transform infrared (FTIR) spectroscopy recorded the infrared spectrum before and after the adsorption in the batch system. X-ray diffractometer was used to find the crystal structure of the oyster shell biocomposite by Rigaku X-ray Diffraction Ultima-Iv, Japan. The pattern of the biocomposite was recorded in the 2θ range between 5° - 80° at 70°C. The morphology of oyster shell biocomposite was determined using a transmission electron microscopy (TEM, LEO912-AB, LEO). Raman spectra of oyster shell biocomposite was analyzed before and after adsorption using Raman spectrometer (i-Raman plus with 785 nm laser excitation). X-ray diffractometer was used to find the crystal structure of the oyster shell biocomposite by Rigaku X-ray Diffraction Ultima-Iv, Japan.

### 2.3 Batch adsorption experiments

The stock solutions of Cd(II) and Pb(II) (1000 mg/L) were prepared respectively for the investigation. All adsorption experiments were performed in three replicates and recorded in average. Isothermal adsorption experiment was carried out on the Cd(II) and Pb(II) stock solution which was diluted respectively according to designed operational process variables for the optimization of parameters using the CCD. The suspensions were oscillated at 150 rpm in a constant temperature oscillator at 25°C. The supernatant was collected and the residual concentrations of metal ions were analyzed by using atomic-absorption spectrophotometry. The influence of the interaction of operational condition of pH, adsorbent dosage, initial concentration and temperature on the adsorption capacity of the modified oyster shell biocomposite for the optimum adsorption of Cd(II) and Pb(II) was evaluated and analyzed using the CCD of the RSM. In the study, optimum adsorption capacity of the biocomposite using dosage between 0.50–3 g/L was applied into the pollutant solutions at a variation interval of initial concentration (20–100) mL. A constant temperature shaker was used to obtain the mixture at 150 rpm for 180 min within 20 to 50°C to achieve the equilibrium. Batch adsorption isotherm and adsorption kinetic adsorption experiment on the adsorption of Cd(II) and Pb(II) was carried out at intervals accordingly (t = 5, 15, 30, 60, 120, 180, 360 and 720 1440 min). The thermodynamic adsorption experiments of the metal ions on the modified oyster shell biocomposite was set at 15, 25 and 35°C, respectively at pH interval of 2–6. The adjustment of the initial pH of adsorption experiments was achieved using 0.1 mol/L HCl and 0.1 mol/L NaOH. The suspensions were finally filtered using 0.45 μm water phase microporous membrane, and the analysis of the concentration of Cd(II) and Pb(II) were measured using Atomic Absorption Spectroscopy (240AAFS, Agilent, USA).


$$Percentage\;removal=R\%=\frac{\left({C_{0}-C}_{e}\right)}{C_{0}}\times 100\%$$ (1)

$$Adsorption\;capacity=q_{e}=\frac{({C_{0}-C}_{e})V}{M}$$ (2)

The removal efficiency of the oyster shell biocomposite for the removal of Cd(II) and Pb(II) was evaluated according to Eqs 1 and 2 respectively.

Where *R* % is the percentage removal, *q*_*e*_ (mg/g) is uptake capacity, *C*_0_ (mg/l) indicates the initial metal ions concentration, *C*_*e*_ (mg/l) is the residual metal ions concentration, V (L) is the solution volume and M (g) is the mass of adsorbent.

#### Modelling of process variables of batch biosorption study

The response surface methodology (RSM) is a statistical tool used for process optimization of operational conditions in order to determine the effect of the process variables on the system. The central composite design (CCD) of the RSM was implemented for the biosorption of Cd(II) and Pb(II) from aqueous solution. The effect of the interaction of the process conditions on the oyster shell biocomposite adsorbent for the adsorption of Cd(II) and Pb(II) were obtained through batch adsorption by investigating the effect of four factors. The process factors were pH, adsorbent dosage, initial concentration and temperature which were conducted under the operational condition according to **Table 2**. Independent variables were denoted as high level (+1), low level (-1) and the centre point (0) using the Design Expert 6.0.4. Corresponding details is depicted in **Table 3** consisting of thirty experimental runs, four central points within a block to determine the effect of the process variables on the removal efficiency of the biocomposite.

**Table 2:** Central composite design analysis for biosorption of Cd^2+^ and Pb^2+^.

Factors	Symbols	Unit	Code	Levels				
				-2	-1	0	+1	+2
pH	A	-	*X*_1_	-	2	4	6	8
Adsorbent dosage	B	g/L	*X*_2_	0.75	0.50	1.75	3.0	4.25
Initial concentration	C	mg/L	*X*_**3**_	10	20	60	100	140
Temperature	^D^	°C	*X*_4_	5	20	35	50	65


$$Y=\beta_{0}+\sum_{i=1}^{K}\beta_{i}X_{i}+\sum_{i=1}^{K}\beta_{ii}{X_{i}}^{2}+\sum_{i=1}^{K-1}\sum_{j=2}^{K}\beta_{ij}X_{i}X_{j}+€$$ (3)

**Table 3:** Independent variables and experimental data for the adsorption of Cd and Pb on oystershell biocomposite.

Runs	A pH	B: Adsorbent b doses g/L	C: Initial concentration mg/L	D: Temperature °C	Y_1_: %Cd Actual	Y_1_: %Cd Predicted	Y_2_: %Pb Observed	Y_2_: %Pb Predicted
1	0.000	-2.000	0.000	0.000	88.40	83.88	60.90	64.56
2	1.000	1.000	1.000	-1.000	23.40	29.64	56.31	52.04
3	0.000	0.000	0.000	-2.000	88.40	93.09	76.40	70.77
4	-1.000	-1.000	-1.000	1.000	91.20	96.38	89.84	91.55
5	0.000	0.000	0.000	2.000	89.20	96.14	89.34	92.66
6	-1.000	1.000	-1.000	-1.000	56.40	46.73	67.89	68.14
7	-1.000	-1.000	1.000	-1.000	89.20	84.18	91.30	86.84
8	-1.000	1.000	1.000	-1.000	87.30	92.29	92.10	95.62
9	0.000	0.000	0.000	0.000	86.40	84.01	49.60	44.87
10	1.000	1.000	-1.000	-1.000	91.2	65.55	23.32	31.54
11	0.000	0.000	0.000	0.000	67.40	67.00	54.30	57.81
12	-1.000	1.000	-1.000	1.000	80.40	76.06	82.30	77.77
13	0.000	0.000	2.000	0.000	77.20	71.95	62.40	64.45
14	1.000	1.000	-1.000	1.000	60.40	58.31	34.70	39.11
15	-1.000	1.000	1.000	1.000	7.40	3.76	62.30	65.35
16	2.000	0.000	0.000	0.000	43.20	47.65	73.20	73.31
17	0.000	0.000	0.000	1.000	89.3	79.75	96.76	87.31
18	1.000	1.000	1.000	1.000	43.2	54.98	81.20	79.24
19	1.000	-1.000	1.000	1.000	60.4	92.56	67.30	62.12
20	1.000	-1.000	-1.000	1.000	98.70	91.11	99.90	102.53
21	1.000	-1.000	1.000	-1.000	78.20	70.66	47.40	49.70
22	0.500	0.000	0.000	0.000	49.50	54.51	78.20	73.35
23	0.000	0.000	0.000	0.000	78.40	74.34	62.50	64.72
24	0.000	0.000	0.000	1.000	28.30	29.83	27.50	22.73
25	0.000	0.000	0.000	0.000	89.30	82.43	92.10	88.31
26	-1.000	-1.000	1.000	1.000	67.90	82.43	76.30	88.31
27	0.000	2.000	0.000	0.000	98.7	82.43	92.10	88.31
28	0.000	0.000	-2.000	0.000	78.2	82.43	76.30	88.31
29	1.000	-1.000	-1.000	-1.000	89.30	82.43	92.10	88.31
30	-1.000	-1.000	-1.000	-1.000	89.30	82.43	92.10	88.31

The response of the model denotes the adsorption capacity Y. Through the relationship of the independent variables, the mathematical expression of metal ions adsorption was established

Where *β*_0_ and *β*_i_ indicate the constant coefficient, while the linear coefficient of the input parameters is represented by X_i_ respectively. *β*_ii_ represent the quadratic coefficient of the input parameter, X_i_, *β*_ij_ indicate the interaction coefficient between the input parameter X_i_ and X_j_ and € represents the error of the model.

#### Point of zero charge of oyster shell biocomposite

To determine the surface charge of the oyster shell biocomposite at different pH values 2–10 [32]. Experiments were conducted using 50 mL 0.1M KCl solution. The solution pH was adjusted using 0.1 M HCl or 0.1 M NaOH concentration. 0.1g of biocomposites were added to each 100 mL of the pH-adjusted solution in an Erlenmeyer flask and the samples were agitated using magnetic shaker for 48 h at room temperature. The final pH (pH_f_) value of supernatant obtained was plotted against the initial pH. The pH at which the curve intercepts the initial (pH_O_) was obtained as the point of zero charge (pH_PZC_).

### Isotherm and kinetic models

#### Adsorption isotherm

Batch adsorption was conducted using an initial concentration of 50 mg/L Cd^2+^ and Pb^2+^ respectively from the prepared solutions. The beaker containing the solution was magnetically stirred at 25°C, and the rotating speed was set at 200 r/min. After then, adsorbent dosage was added to the fully stirred heavy metal solutions for each batch experiment ranging from (3.0–4.50) g. Triplicate samples each in a 250 mL conical flask were placed in an orbital shaker and the mixture was agitated at 150 rpm and samples were withdrawn after 30 min contact time. An approximately 10 mL sample was taken from the solution using a pipette gun and was then vacuum filtered using a 0.45 μm microporous membrane. The heavy metal concentration in the supernatant was then determined using the Atomic Absorption Spectrophotometry (Perkin Elmer; AAS 700 AAS).

In this study, Langmuir, Freundlich and Dubinin-Radushkevich isotherm models were used to fit experimental data to describe the adsorption of Cd (II) and Pb(II) ions adsorbed at the interface of the modified oyster shell biocomposite adsorbent. The models were described according to the following equations [33,34].

Where q_m_ describes the maximum adsorption capacity (mg/g) and K_L_ is the Langmuir constant (L/mg).


$$Langmuir\;model:\;\frac{1}{q_{e}}=\frac{1}{q_{m}}+\frac{1}{{K_{L}q}_{m}C_{e}}$$ (4)

$$Freundlich\;model:\;logq_{e}=logK_{f}+\frac{1}{n}logC_{e}$$ (5)

Where K_f_ is the Freundlich constant ((mg/g) ⋅ (L/mg)1/n) and 1/n denotes the adsorption capacity.


$$Dubinin‐Radushkevich\;isotherm:\;lnq_{e}=lnq_{m}-\beta \varepsilon^{2}$$ (6)

Where β and ε are the model parameters, ε = RTln (1+ $\frac{1}{C_{e}}$), Adsorption process’s free energy was evaluated by formula: E = $\frac{1}{\surd 2\beta }$

### Adsorption kinetics

Adsorption kinetics was conducted using an initial concentration of 50 mg/L Cd^2+^ and Pb^2+^ respectively from the solutions. The beaker containing the solution was magnetically stirred at 25°C, and the rotating speed was set at 200 r/min. After then, adsorbent dosage was added to the fully stirred heavy metal solutions for each kinetic study ranging from 3.0–4.50 g of oyster shell biocomposite. Triplicate samples each in a 250mL conical flask were placed in an orbital shaker and the mixture was agitated at 150 rpm and samples were withdrawn at intervals ranging from 5 to 360 min. An approximately 10 mL sample was taken from the solution using a pipette gun and was then vacuum filtered using a 0.45 μm microporous membrane. The heavy metal concentration in the supernatant was then determined using the Atomic Absorption Spectrophotometry (Perkin Elmer; **AAS** 700 AAS).

Experimental kinetic data were fitted to the Pseudo first—order, the Pseudo second—order, Elovich and Intraparticle diffusion as follows according to Eqs (7–10) respectively.

$${Elovich\;model\;q}_{t}=\frac{1}{\beta }ln\alpha \beta +\frac{1}{\beta }lnt$$ (7)

$$Intraparticle\;diffusion\;model:\;q_{t}=k_{i}t^{0.5}+c$$ (8)

q_e_ denotes equilibrium adsorbate concentration_,_ q_t_ is the amount of adsorbate adsorbed at time t, k_1_ (1/min), k_2_ (g/(mg·min)), and k_3_(mg/(g·min^1/2^)) indicate the adsorption rate constants; and C (mg/g) is the constant of the intraparticle diffusion model. The pseudo-second-order kinetic (Elovich equation) assumed that the actual media surfaces were heterogeneous based on their energy, *q*_*t*_ represents the adsorption capacity, linear relationship with a slope of $\frac{1}{\beta }$ and an intercept of $\frac{1}{\beta }ln\alpha \beta$. where k_i_ describes the intraparticle diffusion constant while C represents the intercept.

### 2.5 Thermodynamics studies

The study of the reaction temperature towards oyster shell adsorption of Cd and Pb from solution was carried out to investigate the adsorption thermodynamics. Accordingly, 2.56 g/L adsorbent dose, initial concentration, 46.76 mg/L at pH 5.5 was conducted for 60 min at different reaction temperature [30–55] ±1°C. The thermodynamic parameters, namely, Gibbs free energy change (Δ*G*°), enthalpy (Δ*H*°), and entropy change (Δ*S*°) were evaluated using equations

$$\Delta G{}^{\circ}=-RT\;ln\;K_{d}$$ (9)

ΔG°=ΔH°−TΔS° (10)

$$\ln K=\frac{\Delta S{}^{\circ}}{R}-\frac{\Delta H{}^{\circ}}{RT}\;$$ (11)

Where R (8.314 J/mol.K) represents the universal gas constant, T (°K) denotes the absolute temperature and K_d_ defines the dissociation constant.

### Desorption and reusability studies

For recyclability study, the adsorbed Cd and Pb metals on the biocomposites were eluted with 0.1 M HCl solution, the adsorbents were further washed with deionized water. The analytes were oven dried for 24 h to obtain a regenerated biocomposite which was reused for successive cycles of adsorption experiments. After then, next cycles of successive adsorbent experiments were conducted using the regenerated adsorbents. A total of five cycles of batch adsorption experiments were conducted on the desorbed biocomposite at room temperature under optimum condition above.

## 3. Results and discussion

### 3.1. Characterization of oyster shell biocomposite

**Fig 1:**
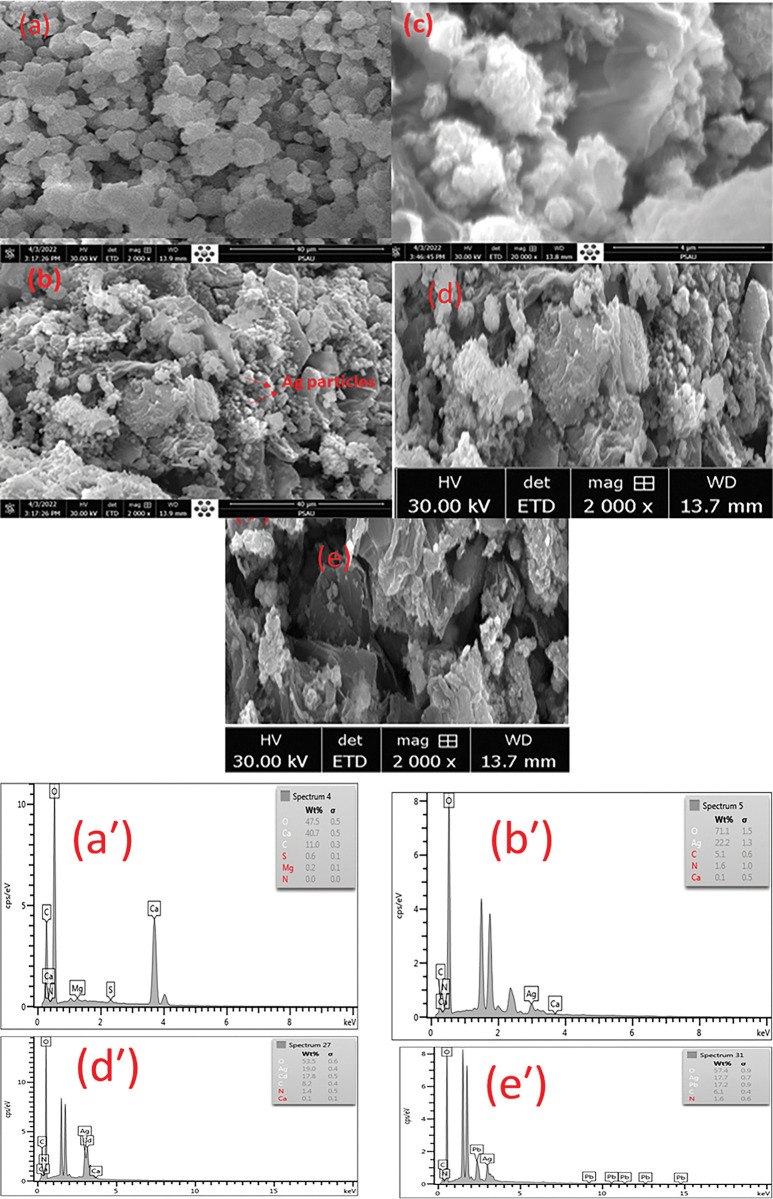
SEM images of (a) Oyster Shell carbon material (b and c) Acid treated oyster shell biocomposite; EDS Images (aˈ-eˈ)Oyster shell biocomposite.

The structural analysis was carried out to determine the surface morphology of oyster shell powder and the modified oyster shell biocomposite. The morphology of the oyster shell powder and oyster shell biocomposites were presented in Fig 1A and 1B respectively. From Fig 1(A), aggregation of irregular and rough surface area were the predominant features on the oyster shell powder structure with noticeable holes in most parts of the surface proving that oyster shell has a porous surface that provided surface for the incorporation of silver ions particles in the biocomposite structure. The microstructures of the oyster shells suggest that heat was not evenly distributed across the shells during the calcination process. However, Fig 1(B) indicated that silver particles were deposited onto the surface of biocomposite thereby creating a fine and coarse textural surface morphology. Fig 1(C) shows high magnification of Fig 1(B) with particle size 4μm. The introduction of Ag particles to oyster shell powder could decrease the aggregation and provide the oyster shell biocomposite more adsorbing sites for Cd(II) and Pb(II) metal ions. As shown in Fig 1D and 1E, denoting the surface morphology of the modified oyster shell biocomposite after the adsorption of Cd(II) and Pb(II), it was revealed that the microstructure became tightly packed which indicated that the incorporation of Ag particles clusters were homogenously distributed on the oyster shell biocomposite. Thus, enabling the adsorption of Cd(II) and Pb(II) ions on its surface. The morphologies were similar to that of the functional biocomposite in previous report [35]. The elemental composition of the oyster shell powder and the modified oyster shell biocomposite from the EDS analysis according to **Fig 1Aˈ and 1**B**ˈ** respectively revealed that Ca and O noticeably have high percent elemental composition. It was noticed in **Fig 1Dˈ** that the surface morphology revealed the presence of Ag particles on its surface suggesting that the aggregation of the particles on the oyster shell biocomposite may be as a result of the irregular pore structures created during calcination.

The elemental composition of the oyster shell powder and the modified oyster shell biocomposite from the EDS analysis according to **Fig 1E and 1D** respectively revealed that Ca and O noticeably have high percent elemental composition. It was noticed in **Fig 1E** that the surface morphology denoted the incorporation of Ag particles on the surface of the oyster shell biocomposite.

The adsorption capacity of the modified oyster shell biocomposite from the EDX spectrum in **Fig 1Dˈ and 1Eˈ** showed that the surface of the biocomposite provided binding properties for the adsorption of Cd(II) and Pb(II) compared to the neat modified oyster shell biocomposite before adsorption as indicated in the EDX spectra before adsorption in **Fig 1A** The adsorbed Cd(II) and Pb(II) ions were detected respectively on the modified oyster shell biocomposite.

The specific surface area of the modified oyster shell powder determined by N_2_ adsorption at 77 K showed that the BET surface was 9.1476 m^2^/g was higher than the BET specific surface area of oyster shell powder and ion imprinted oyster shell 4.64 m^2^/ g and 5.63 m^2^ /g respectively in the study of Wu et al. [36]. The average pore diameter was 5.1803 nm indicating a mesoporous pore surface properties [37]. The specific surface area and the pore size slightly decreased as a result of the aggregation of silver particles on the surface of the modified oyster shell powder without change in the mesoporous structure [38]. This is reflected by the values of S_BET_ and pore diameter of 7.1017 m^2^/g and 5.0951 nm respectively. The pore volumes of acid treated oyster shell powder and the oyster shell biocomposite are evaluated (p/p_0_ = 0.99) to be 0.018657 and 0.023572 cm^3^/g. The pore volume of ultrafine oyster shell in the study of Su et al. depicted pore volumes to be 0.009, 0.011 and 0.015 cm^3^/g [39]. The pore size distribution indicates that oyster shell biocomposite have great numbers of micropores and mesopores which enhances its adsorption capacity.

**Fig 2:**
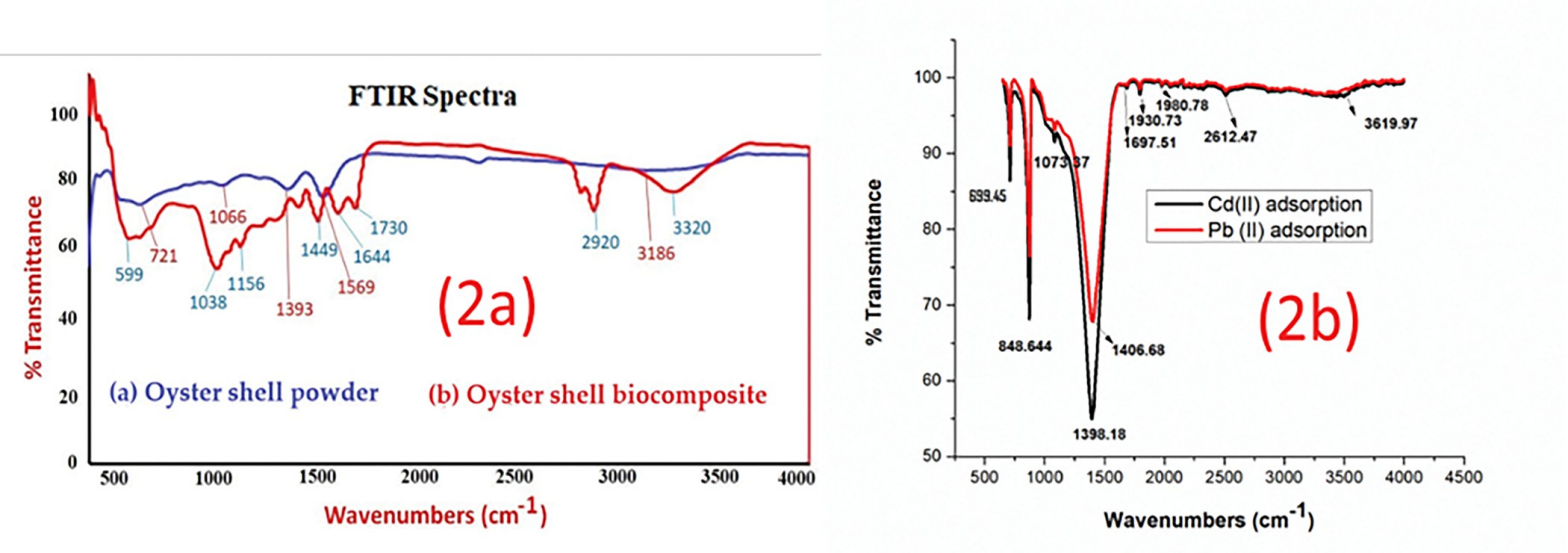
FT-IR spectra (a) Oyster shell powder; Oyster shell biocomposite (b) Cd and Pb adsorption on oyster shell biocomposite.

The functional properties using the FTIR spectra data was obtained for the analysis of the associated functional groups on the surface of the oyster shell powder and oyster shell biocomposite. As indicated from **Fig 2(A),** the appearance of broad peak near 3186 cm^-1^ may be due to the presence of hydroxyl group (–OH) group on the adsorbent surface. The peak around the wavelength of 1569 cm^-1^ was attributed to the presence of–C = C-H groups. A peak appeared near 1393 cm^-1^ which corresponds to the presence of alkane (C-H) bending vibration. In addition, the peak assigned near 1066 cm^-1^ was attributed to the presence of C-O stretching vibration. After activation with acid and Ag particles, characteristic peaks denoting hydroxyl (-OH) group and C-H stretching vibration can also be observed in Fig 2(B) near wavelength 3320 cm^-1^ and 2920 cm^-1^ respectively. A peak around 1730 cm^-1^ was assigned for (C = O) group of carboxylic group of carbonate ion [40], indicating the presence of CaCO_3_ in the oyster shell biocomposite structure. The wavelength around 1156 cm^−1^, 1449 cm^−1^ and 1644 cm^−1^ were characteristic peaks denoting the presence of Ag particles as an integral component of the oyster shell biocomposite. Similar peaks have been reported by Babu and Antony [41]. An intense peak appeared around 599 cm^−1^ which can be assigned for the Ag-O stretching vibration. Similar result has been reported by Khalir et al. 2020 [42].

These additional peaks confirmed that due to the acid and silver nitrate treatment subjected to the neat oyster shell powder, it was revealed that the surface properties of the oyster shell biocomposite were exposed to the carboxylic (-COOH), hydroxyl (-OH), and metal oxide (Ag-O) functional groups incorporated onto the surface of oyster shell biocomposite which influenced the binding and adsorption of the metal ions to its surface.

In Fig 2b, the functional properties of the modified oyster shell biocomposite was examined after the adsorption of Cd(II) and Pb(II) ions. The peak of 3619.97 cm^-1^ was assigned to stretching vibration of dissociative O-H from Ca(OH)_2_,which was transformed by the reaction of CaO as heating temperature increased Lian et al. [43]. The strong absorption band at 1612 cm^−1^ corresponded to the C = O stretching vibration of the CO_3_^2−^ molecule in CaCO_3_ indicating the presence of CaCO_3_ in the oyster shell biocomposite which significantly influenced adsorption of Cd(II) and Pb(II) ions [44]. Other bands feature which denote CH out of plane bending vibrations in substituted ethylinic systems was assigned at band 689.45 cm^-1^. The band at 1073.37 cm^-1^ was attributed to C-O-H functional group in the oyster shell biocomposite. The bands at 1406 cm^-1^ and 1697.51 cm^-1^ denote CH stretching vibration of the CH_2_ groups and C = C bending vibrations respectively. The incorporation of silver nitrate occurred around 3320 cm^-1^ band which signified the presence of N-H groups on the surface of the oyster shell biocomposite. The band at 2920 cm^-1^ indicated the addition of citric acid which was attributed to the C-H groups and carboxyl groups on the surface. In addition, citric acid from the lemon juice contributed alkanes and ketones groups. The role of the acid was to improve the dehydration property and to increase active functional groups on the surface [45].

**Fig 3:**
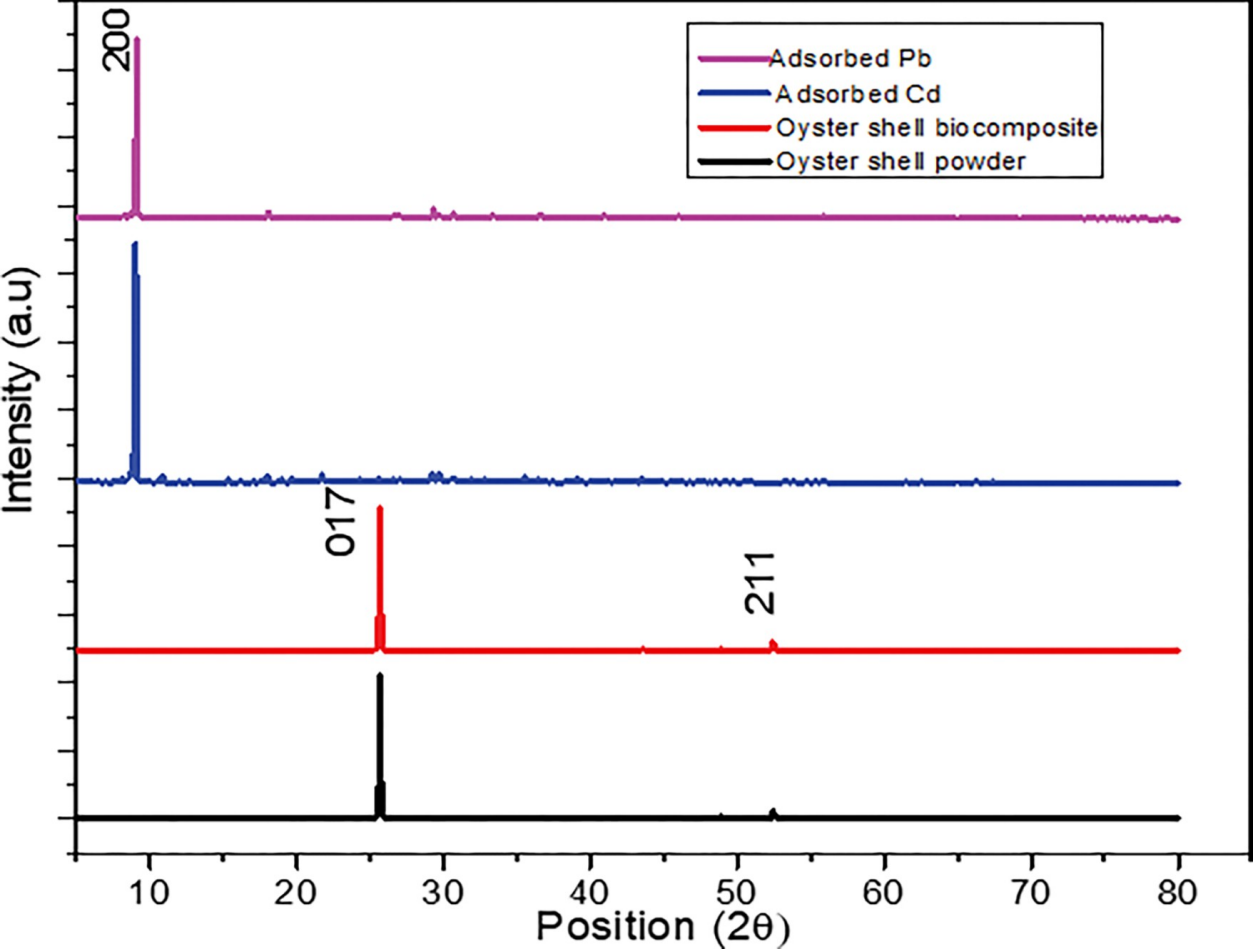
XRD pattern of oyster shell powder and Oyster shell biocomposite for Cd and Pb adsorption.

The crystalline structures of both the oyster shell powder and the modified oyster shell biocomposite (Fig 3) were examined using XRD diffraction spectra before and after adsorption of Cd(II) and Pb(II). The X-ray diffraction patterns (XRD) was obtained in the range of 5–60° as shown in **Fig 7.** The main characteristics of oyster shell powder and modified oyster shell biocomposite was obtained at 2Ѳ of 25° was assigned to 017 which indicated the presence of calcium carbonate polymorph calcite phase (JCPDS card no. 05–0586.11). The XRD diffraction peak revealed that the adsorption of Cd(II) and Pb(II) occurred at 2Ѳ of about 7° which corresponds to (200) hkL plane. This agrees with previous study by Nyirenda et al. [46].

**Fig 4:**
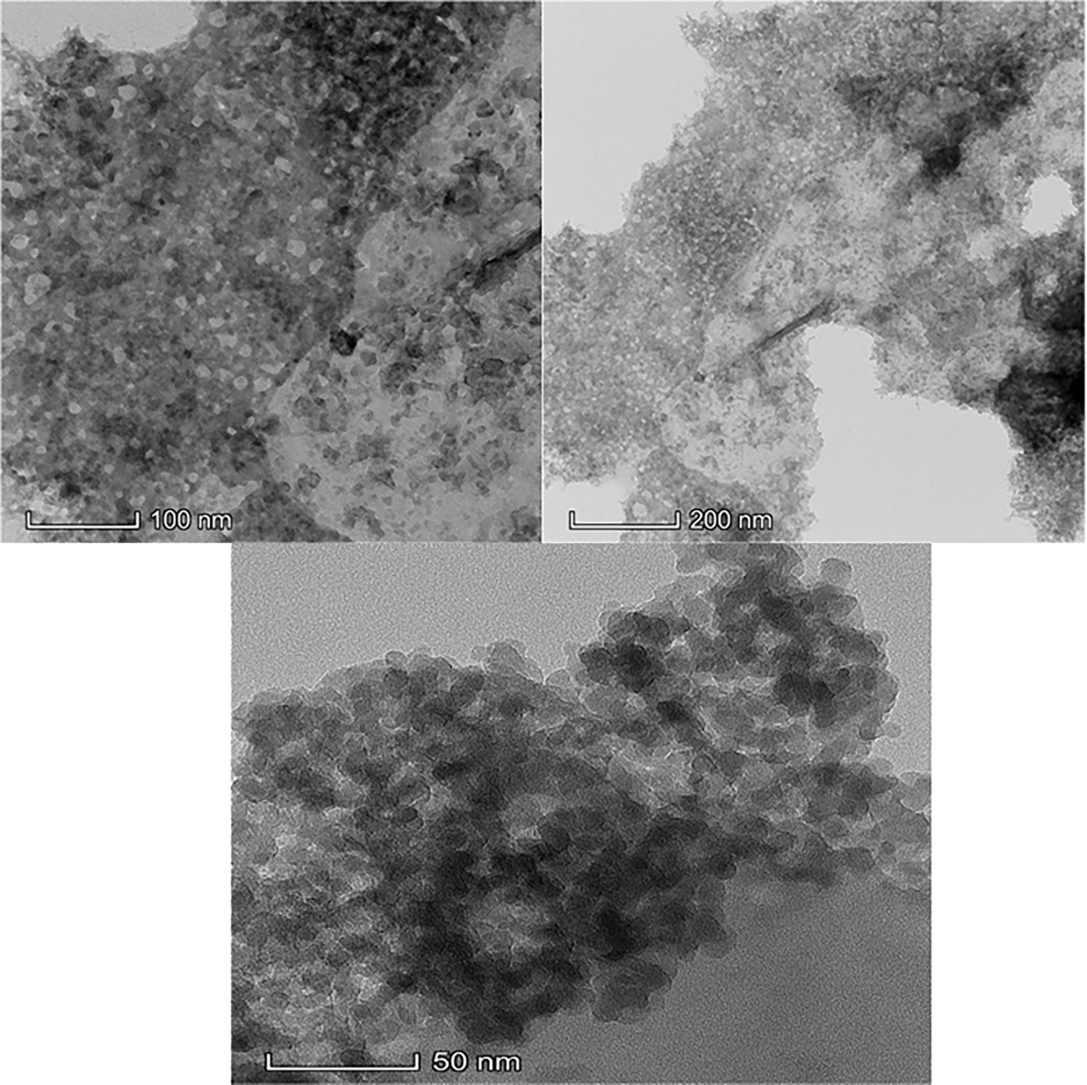
TEM of oyster shell biocomposite before and after adsorption of Cd and Pb.

Fig 4 Denotes the TEM images before and after of Cd and Pb adsorption. The morphological structure of the oyster shell biocomposite appears coarse with pores distribution on the surface creating active sites for adsorption. The dense surface geometry was as a result of the incorporation of Ag particles on the surface area of the oyster shell powder forming the biocomposite After adsorption, the pore surface appeared dense, and closely packed indicating that Cd and Pb metals were effectively incorporated within the voids of the adsorbent. (Fig 2B–2c). It is noticed that this crystal interfacial structure provided a binding surface for the attachment of the metal ions during adsorption.

**Fig 5:**
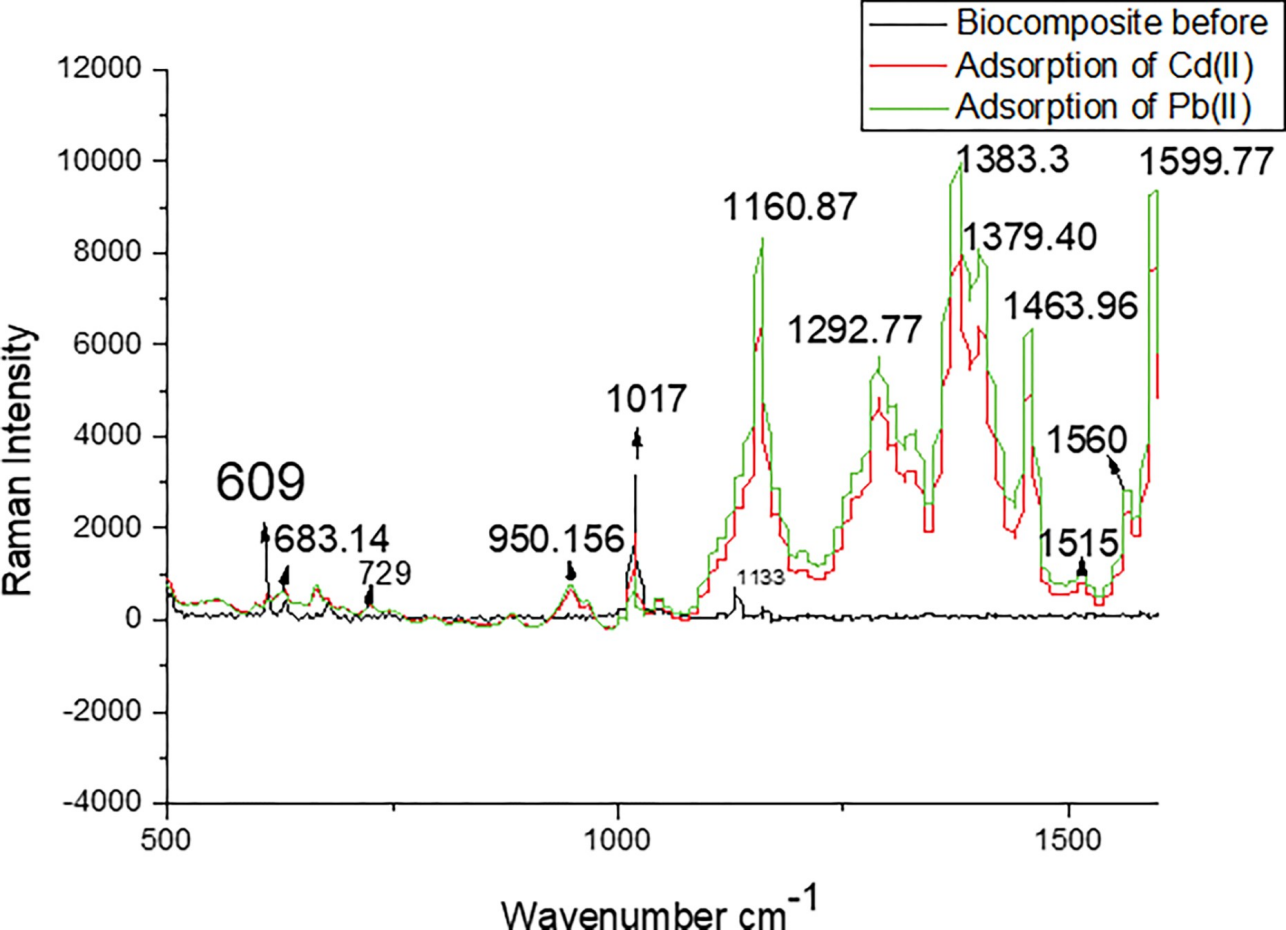
Raman spectra of oyster shell biocomposite before and after Cd and Pb adsorption.

**Fig 5** from the Raman spectra shows the super imposition between spectra lines at bands at 609, 683.14, 729 and 1017 cm^-1^ indicating the presence of calcite polymorph of CaCO_3_ present in oyster shell biocomposite before and after adsorption. A noticeable disappearance of peaks was achieved on the oyster shell biocomposite before adsorption of Cd(II) and Pb (II) ions. However increase in spectrum was noticed between 1292.72–1463.96 cm^-1^ suggesting that these spectra prominently influenced the adsorption of Cd(II) and Pb(II) by the occupation of available sites and functional groups. The Raman spectra at around 1560–1599.77 cm^-1^ was assigned to the carboxylate asymmetric stretching band. This asymmetric vibration indicated the interaction between the carboxylate groups on the oyster shell composite and the Ag (II) ions resulting in the effective adsorption of Cd (II) and Pb(II) ions [47]. No noticeable spectra bands were recorded at this range for the oyster shell biocomposite indicating that the active functional groups may have been occupied by the biocomposite after adsorption of Cd(II) and Pb(II) ions.

### 3.2 Batch adsorption experiments

Batch adsorption experiments were conducted under the operational limits of the experimental design as shown in **Table 2.** The result of the experimental values and the predicted values for the adsorption of Cd and Pb on the oyster shell biocomposite is presented in **Table 3.**

**Fig 6:**
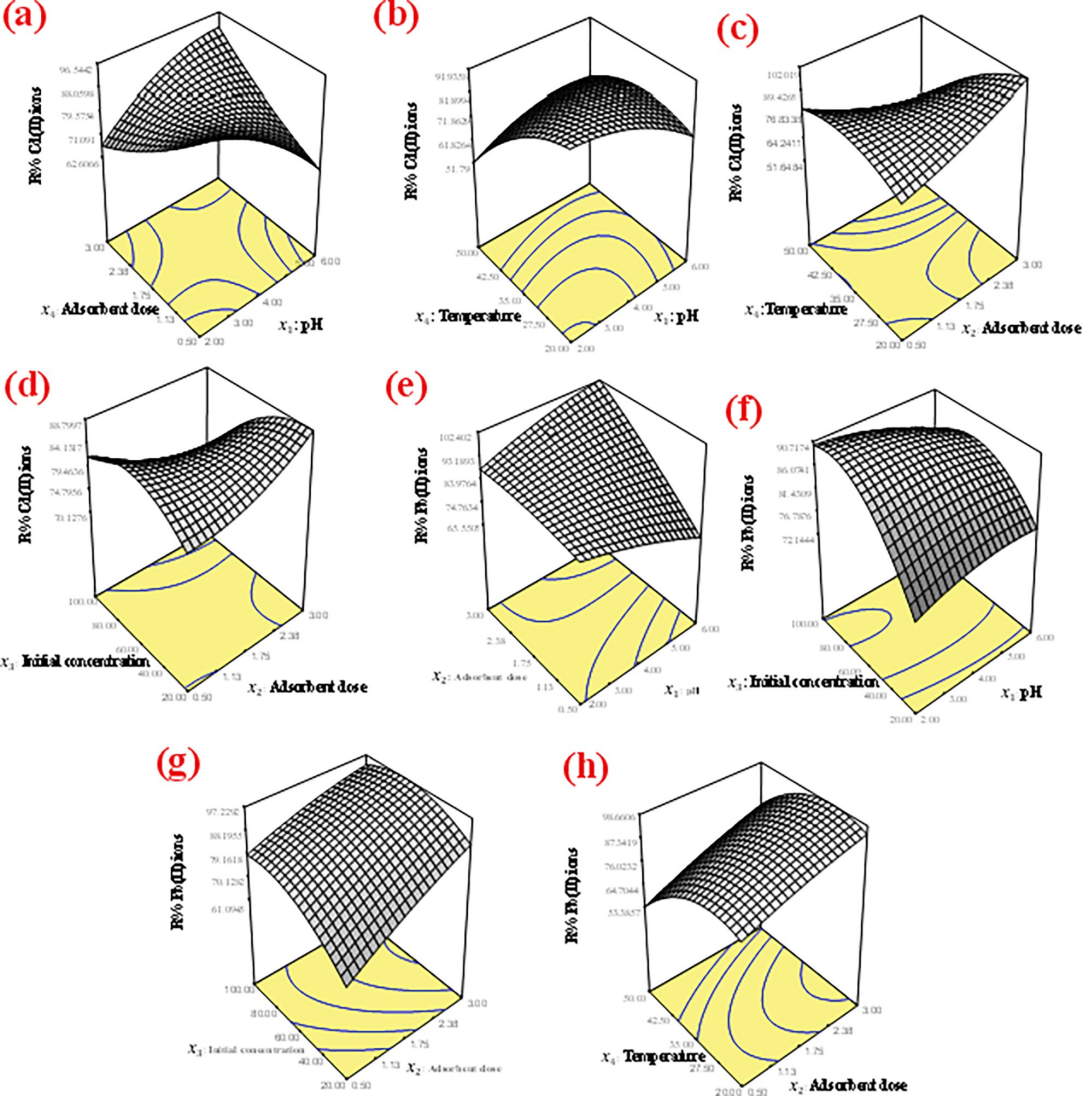
3D surface plot for the interaction of **(a)** pH and adsorbent dosage **(b)** pH and temperature **(c)** adsorbent dose and temperature **(d)** Adsorbent dose and initial concentration for Cd(II) ion removal **(e)** pH and adsorbent dosage **(f)** pH and Initial concentration **(g)** Adsorbent and initial concentration **(h)** Adsorbent dose and temperature for Pb(II) ion removal on oyster shell biocomposite.

The interactive effect of the process variables was analyzed using 3D response surface plot in **Fig 6.**

The **Fig 6A** represents the interaction of adsorbent dosage and pH. It was revealed that increase in the adsorbent mass favoured maximum removal of Cd(II) ions from solution. It is clear that the rapid increase of the percent removal of the metals was achieved with increase in the dosage of the adsorbents which can be attributed to the availability of the exchangeable sites for adsorption. Moreover, the percentage of metal ion adsorption on adsorbent determines the adsorption capacity of the adsorbent for various metal ions. Increase in pH could have influence on the adsorption capacity as a result of the formation of soluble hydroxyl complexes. The effect of the interaction of pH and temperature (**Fig 6B**) increased the adsorption efficiency of the biocomposite for the adsorption of Cd(II) ions. This was reflected by the positive correlation with the response as the pH and adsorption temperature increased. The increase in adsorption capacity of adsorbent with temperature signifies an endothermic process. The above results were further elaborated by the various thermodynamic parameters evaluated for adsorption of metal ions. The increase in adsorbent dosage in the interaction of temperature and adsorbent dosage (**Fig 6C**) further provided available sites and elevated temperature increased diffusion pathway on the surface of the adsorbent. The interactive effect of initial concentration and adsorbent doses is indicated in **Fig 6D**. Higher concentration of the adsorbate tends to be relative to the available adsorption sites [48]. Therefore, the percent removal of the metal ions depended on the initial concentration which decreases as the initial concentration increases. Increase in adsorbent dosage provided additional available sites and the tendency for increase in metal ions on the adsorbent surface. The interactive effect of the process variables for the adsorption Pb(II) ions is illustrated in **Fig 6E–6h**. pH and adsorbent dosage increase which favoured the increased adsorption capacity of the biocomposite for the metal ions adsorption (**Fig 6E**). This trend was also revealed in the interaction of initial concentration and pH. Increase in the initial concentration requires more available sites for the adsorption of metal ions, this resulted to decrease in removal efficiency of Pb(II) ions. pH influenced adsorption of metal ion on the adsorbent surface (**Fig 6F**). The removal capacity was further enhanced by the increase in adsorbent dosage which provided additional sites for metal adsorption as the initial concentration was increased (**Fig 6G**). In addition, increase in temperature and adsorbent dosage favoured increased adsorption of metal ion on the biocomposite. As the adsorbent dose was increased from 0.50 to 3.0 g/L, good interactive influence of the process parameter exhibited remarkable influence in the removal efficiency (**Fig 6H**). At lower dose (0.50 g/L), there is less affinity for the diffusion pathway of metal ions due to the limited number of available binding sites; therefore, a low percentage removal was achieved. A progressive increase in adsorbent dose until 3.0 g/L, would result to a corresponding increase in percentage removal due to more adsorption sites that are available for both metal uptake [48].

#### 3.2.2. Diagnostic plot

The diagnostic analysis was used to validate the significance and adequacy of the model for the interpretation of the effect of the process variables for the removal of the responses. The design expert integrates the process factors at tunable levels resulting to the responses outcome. The diagnostic plots are shown in **Fig 4A–4d.**

**Fig 7:**
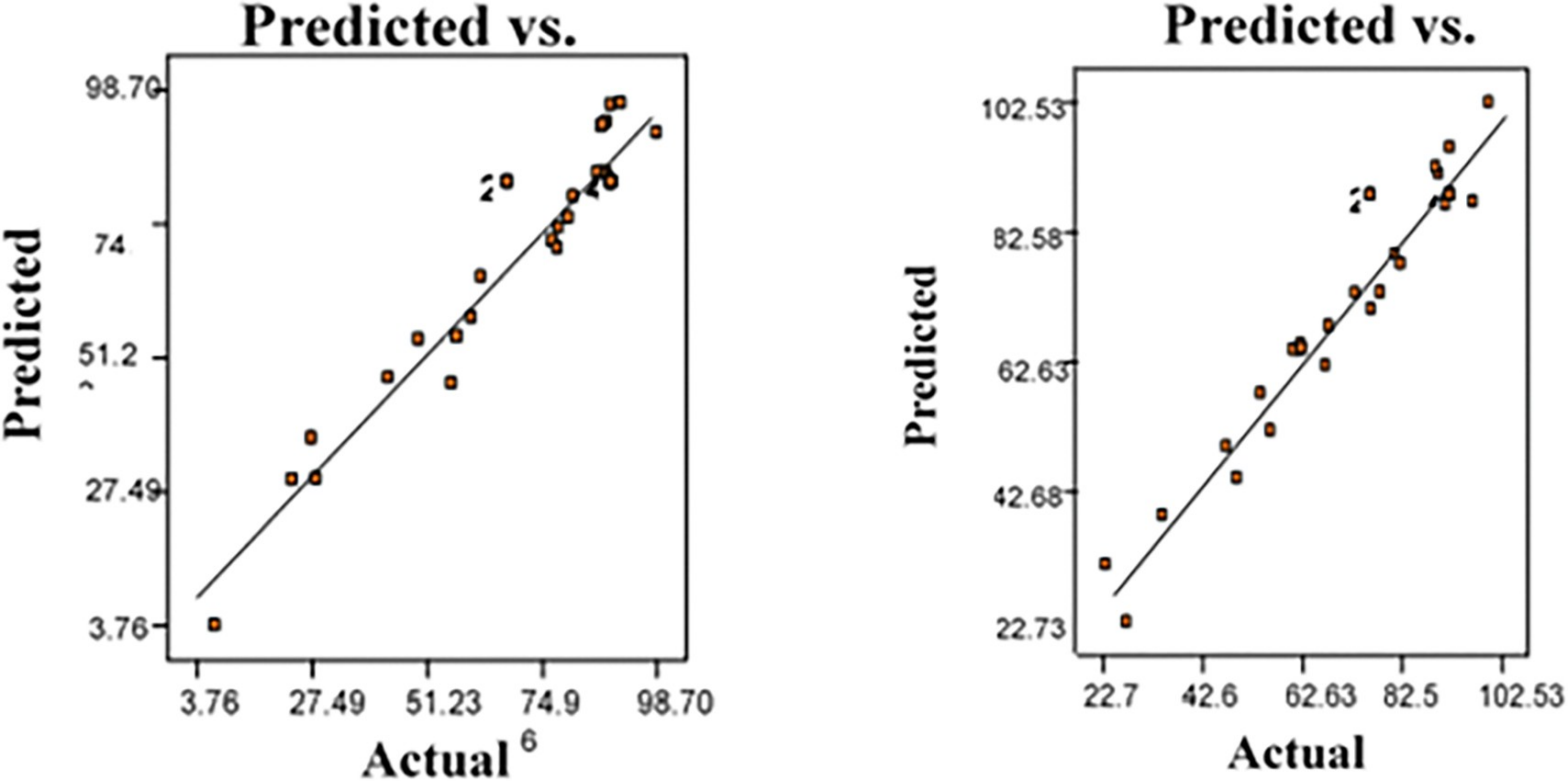
Plot of (**a** and **b**) predicted and actual value of normal distribution and residual value.

In **Fig 7A and 7B**, the distribution of actual and predicted points was closely aligned to the linear plot (y = x), indicating that the disparity of the actual and predicted results was mostly not significant to the responses outcome. In general, all the process factors exhibited a reasonable impact on the responses. For instance, optimum zones could be reached out at nearly 90% attributed to pH, low concentration and large dosage and at increase in temperature.

### 3.3. Statistical analysis of regression model

The central composite design (CCD) of response surface was used to evaluate the statistical significance of the effect of the interaction of the operational process variables for the removal of the responses which were Cd(II) and Pb(II) ions on oyster shell biocomposite. The result of the combined effect of four process variables was evaluated from 30 experimental runs (Table 4).

**Table 4:** ANOVA of response second order quadratic model for percentage removal of Cd(II) ions on oyster shell biocomposite.

Source	Sum of squares	DF	Square values	F-value	P-Value	Remarks
Model	15416.68	14	1101.19	12.90	< 0.0001	Significant
*X*_1_	115.76	1	115.76	1.36	0.2624	Not Significant
*X*_2_	3.15	1	3.15	0.037	0.8502	Not Significant
*X*_3_	391.23	1	391.23	4.58	< 0.0491	Significant
*X*_4_	2972.60	1	2972.60	34.82	< 0.0001	Significant
*X*_1_^2^	505.76	1	505.76	5.92	< 0.0279	Significant
*X*_2_^2^	152.84	1	152.84	1.79	0.2008	Not Significant
*X*_3_^2^	681.13	1	681.13	7.98	< 0.0128	Significant
*X*_4_^2^	1592.41	1	1592.41	18.65	< 0.0006	Significant
*X*_1_ *X*_2_	3309.13	1	3309.13	38.76	< 0.0001	Significant
*X*_1_ *X*_3_	23.28	1	23.28	0.27	0.6092	Not Significant
*X*_1_ *X*_4_	1279.85	1	1279.85	14.99	< 0.0015	Significant
*X*_2_ *X*_3_	448.38	1	448.38	5.25	< 0.0368	Significant
*X*_2_ *X*_4_	3161.25	1	3161.25	37.03	< 0.0001	Significant
*X*_3_ *X*_4_	591.71	1	591.71	6.93	< 0.0188	Significant

**Regression equation in terms of coded factor for Cd(II) ion removal**

$$\%\mathrm{Removal}\;(Y_{1})=+82.43-2.59\;X_{1}-0.36X_{2}-4.04X_{3}-11.13X_{4}-5.57\;{X_{1}}^{2}+2.35\;{X_{2}}^{2}-4.96\;{X_{3}}^{2}-7.59\;{X_{4}}^{2}+14.38X_{1}X_{2}+1.21X_{1}X_{3}+8.94X_{1}X_{4}-5.29X_{2}X_{3}-14.06X_{2}X_{4}-6.08X_{3}X_{4}$$ (12)

**Regression equation in terms of coded factor for Pb(II) ion removal**

$$\%\mathrm{Removal}\;(Y_{2})=+88.31-1.14X_{1}-10.1X_{2}+5.91X_{3}-10.50X_{4}-1.50{X_{2}}^{2}-6.70{X_{3}}^{2}-11.15{X_{4}}^{2}+8.32X_{1}X_{2}-3.00X_{1}X_{3}-0.20X_{1}X_{4}-3.01X_{2}X_{3}+1.68X_{2}X_{4}-{2.13X}_{3}X_{4}$$ (13)

The results of the responses from the experimental design were analyzed using analysis of variance (ANOVA) (**Table 5)**.

**Table 5:** ANOVA of response second order quadratic model for percentage removal of Pb(II) ions on oyster shell biocomposite.

Source	Sum of squares	DF	Square values	F-value	P-Value	Remarks
Model	11736.89	14	11736.89	838.35	< 0.0001	Significant
*X*_1_	22.47	1	22.47	0.42	0.5247	Not Significant
*X*_2_	2449.86	1	2449.86	46.25	< 0.0001	Significant
*X*_3_	838.51	1	838.51	15.83	< 0.0012	Significant
*X*_4_	2645.16	1	2645.16	49.94	< 0.0001	Significant
*X*_1_^2^	46.93	1	46.93	5.92	0.0279	Significant
*X*_2_^2^	152.84	1	152.84	1.79	0.2008	Not Significant
*X*_3_^2^	1240.30	1	1240.30	23.42	< 0.0002	Significant
*X*_4_^2^	3436.69	1	3436.69	64.88	< 0.0001	Significant
*X*_1_ *X*_2_	1108.22	1	1108.22	20.92	< 0.0004	Significant
*X*_1_ *X*_3_	144.12	1	144.12	2.72	0.1198	Not Significant
*X*_1_ *X*_4_	0.67	1	0.67	0.013	0.9118	Not Significant
*X*_2_ *X*_3_	0.1190	1	0.1190	2.73	0.1190	Not Significant
*X*_2_ *X*_4_	45.16	1	45.16	0.85	0.3704	Not Significant
*X*_3_ *X*_4_	72.68	1	72.68	1.37	0.2597	Not Significant

Accordingly, the F-value indicated coefficient of 12.90 for the removal of Cd(II) ions. Meanwhile the P-value of the model was found to be very low (P < 0.0001) [33]. The lack of fit (0.8040) was not significant which revealed that higher probability of the model adequately fitted data [49]. The model indicated that for single linear variable according to ANOVA suggests that the effect of temperature significantly influenced the removal capacity of the oyster shell biocomposite for the adsorption of Cd(II) ions (P < 00001, SS = 2972.60, F = 34.82). The next most significant linear process variable on the response was the effect of initial concentration which was achieved at equivalent (P < 0.049, SS = 391.23, F = 4.58). The linear effect of pH (P = 0.2624) and adsorbent dosage (P = 0.8502) exhibited less significance on the response. The quadratic coefficients of pH, adsorbent dosage and temperature enhanced the removal of Cd(II) ions at (P < 0.03). Similarly, the F-value (15.83) and the corresponding P-value (P<0.0001) indicated that the model was well predicted for the removal of Pb(II) ions from solution. Also the outcome response suggested that the four process variables exhibited significant influence on the adsorption capacity of the oyster shell biocomposite. The effect of linear process optimization revealed that adsorbent dosage, initial concentration and temperature were significant factors influencing high adsorption efficiency (P<0.001). A measure of the signal to noise ratio is determined by the adequate (Adeq) precision which is desirable if the value is greater than 4 [50]. The values of the Adeq precision for both responses Cd(II) ions Pb(II) ions were 14.176 and 15.507 respectively which reflected adequate signal. A model is most suitable for the prediction of response depending on the closeness of correlation coefficient R^2^ to 1.0000 [51]. The interpretation of the statistical model as contributed by the process variables indicated that R^2^ value of 0.9233 (Cd(II)) ions and 0.9366 (Pb(II)) ions was obtained. This implied that the four process variables significantly influenced the adsorption process. The Adj R^2^ (0.8774) and (0.8517) was in reasonable agreement with the values of R^2^ for the removal of Cd (II) and Pb(II) ions respectively which suggested a good agreement between experimental data and predicted data (**Table 6**).

**Table 6:** ANOVA for response second order quadratic model for percentage removal of Cd(II) and Pb(II) ions on oyster shell biocomposite.

Values	Percent removal of Cd(II) ion	Percent removal of Pb(II) ion
*R* ^2^	0.9233	0.9366
*R*^2^ Adj	0.8517	0.8774
Pred *R*^2^	0.6931	0.7686
Adeq Precision	14.176	15.507
Cor Total	16697.26	12531.43
Lack of fit	0.8040	0.7095
Std Dev	9.24	7.28

### 3.4. Validation of model

The statistical significance of the second order model for the interpretation of the effect of the process variables were determined through a confirmation test to validate the proposed optimized results of the CCD using RSM. Accordingly, the difference of the predicted and actual data based on the relative errors were insignificant implying that the model was very suitable for the analysis of the responses under the operational condition of the studied parameters. At practical outcome, 98.7% of Cd(II) was optimally adsorbed. On the other hand, 99.9% removal of Pb (II) ion was contributed by the four process variables at (pH = 5.57, adsorbent dosage = 2.53 g/L, initial concentration = 46.76 mg/L, temperature = 28.48°C). The value of desirability coefficient of 1.0000 was achieved which affirmed that the high suitability of the second order model was attainable for the interpretation of the effect of the process variables for the removal of the responses on the oyster shell biocomposite. This indicates that the experiments conducted based on CCD can be used effectively to optimize the parameters.

### 3.5. Adsorption isotherm adsorption kinetics and thermodynamics

#### 3.5.1 Adsorption isotherm studies

**Fig 8:**
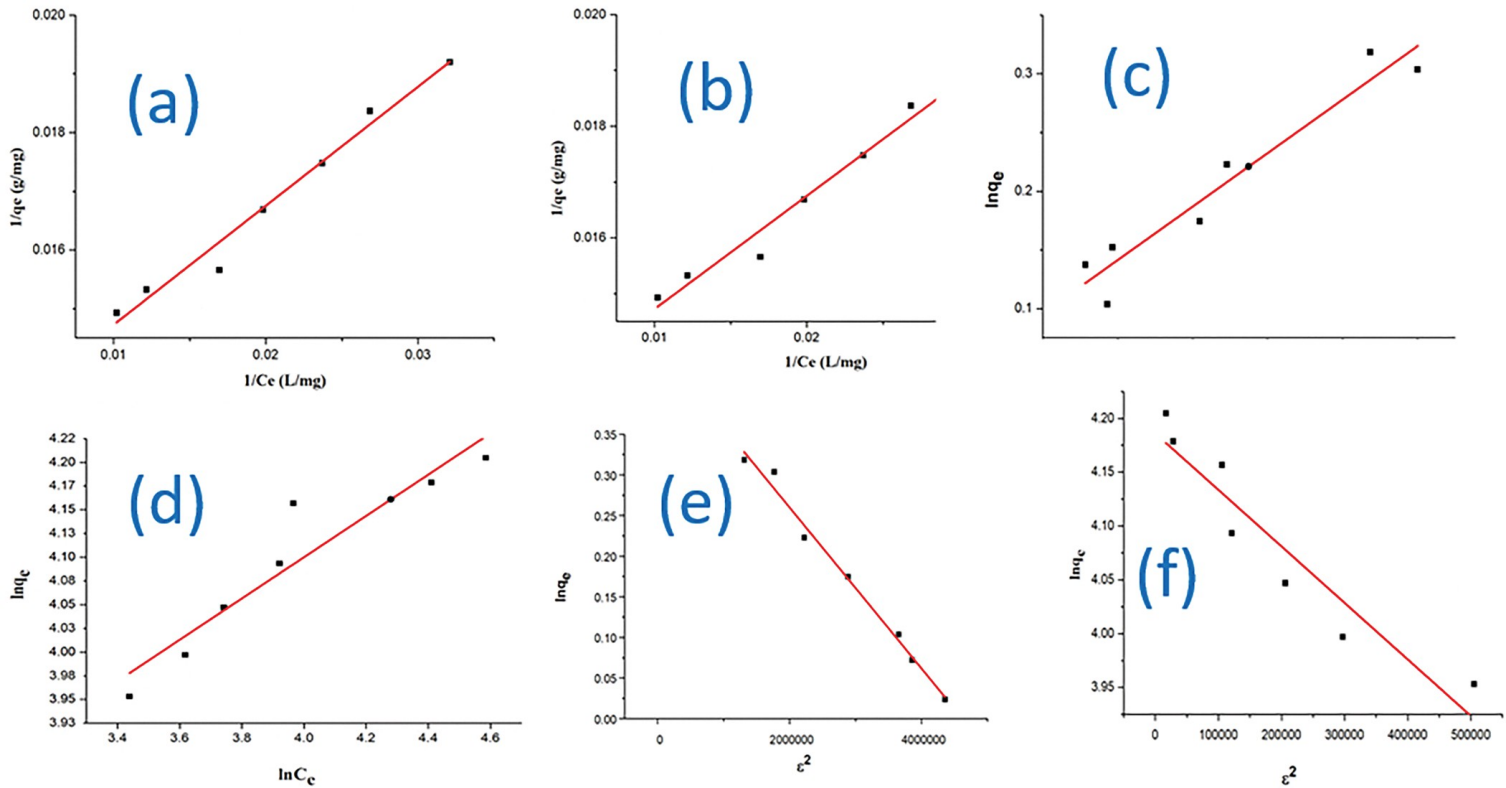
Langmuir Isotherm model for the adsorption of **(a)** Cd(II) **(b)** Pb(II) ions; Freundlich Isotherm model for the adsorption **(c)** Cd(II) **(d)** Pb(II) ions; Dubinin-Raduskevich Isotherm plot for the adsorption of **(e)** Cd(II) **(f)** Pb(II) ions on Oyster shell biocomposite.

To investigate the interaction of Cd(II) and Pb(II) adsorption on the oyster shell biocomposite, experimental data of batch adsorption were fitted to isotherm models which were Langmuir, Freundlich and the Dubinin-Radushkevich isotherm. The fitting effect of the data on the models represented by the correlation coefficient is shown Fig 8A–8F. The parameters were presented in Table 7. Langmuir model gives the assumption that the surface of adsorbent exhibits uniform binding sites with equivalent adsorption energy. Freundlich model describes a multiple-layers adsorption on heterogeneous surface. Meanwhile, the Dubinin-Radushkevich isotherm model combines the description of Langmuir and Freundlich model illustrating uniform and non-uniform adsorption sites [34]. The maximum adsorption capacity (*q*_*max*_) as represented by the Langmuir isotherm model was slightly close to the experimental value with high correlation coefficient compared to Freundlich and the Dubinin-Radushkevich isotherm. This was indicated in the value of *q*_*max*_ as 97.54 and 78.989mg/g for Cd(II) and Pb(II) ions respectively. The linear fitting Langmuir isotherm suitably described the uptake capacity of the oyster shell biocomposite for the adsorption of Cd(II) and Pb(II) ions from solution.

**Table 7:** Linear fitting of Isotherm models parameters on Oyster shell biocomposite.

Langmuir					Freundlich			Dubinin-Radushkevich		
Heavy metal ion	*q*_*max*_ (mg/g)	*q*_*max*_ (mg/g) exp	*K*_*L*_ (L/mg)	*R* ^2^	*K*_*F*_ (mg/g(L/mg)^1/n^)	n	*R* ^2^	*q*_*max*_ (mg/g)	E (KJ/mol)	*R* ^2^
Cd(II)	97.54	86.73±0.074	0.71563±0.021	0.9894	3.01±0.083	2.098±0.11	0.91689	15.81±0.087	2247.3±0.014	0.9870
Pb(II)	78.989	79.81±0.0028	1.595±0.013	0.9746	2.674±0.096	1.593±0.03	0.87111	13.090±0.074	975.90±0.019	0.8811

#### 3.5.2. Adsorption kinetic studies

**Fig 9:**
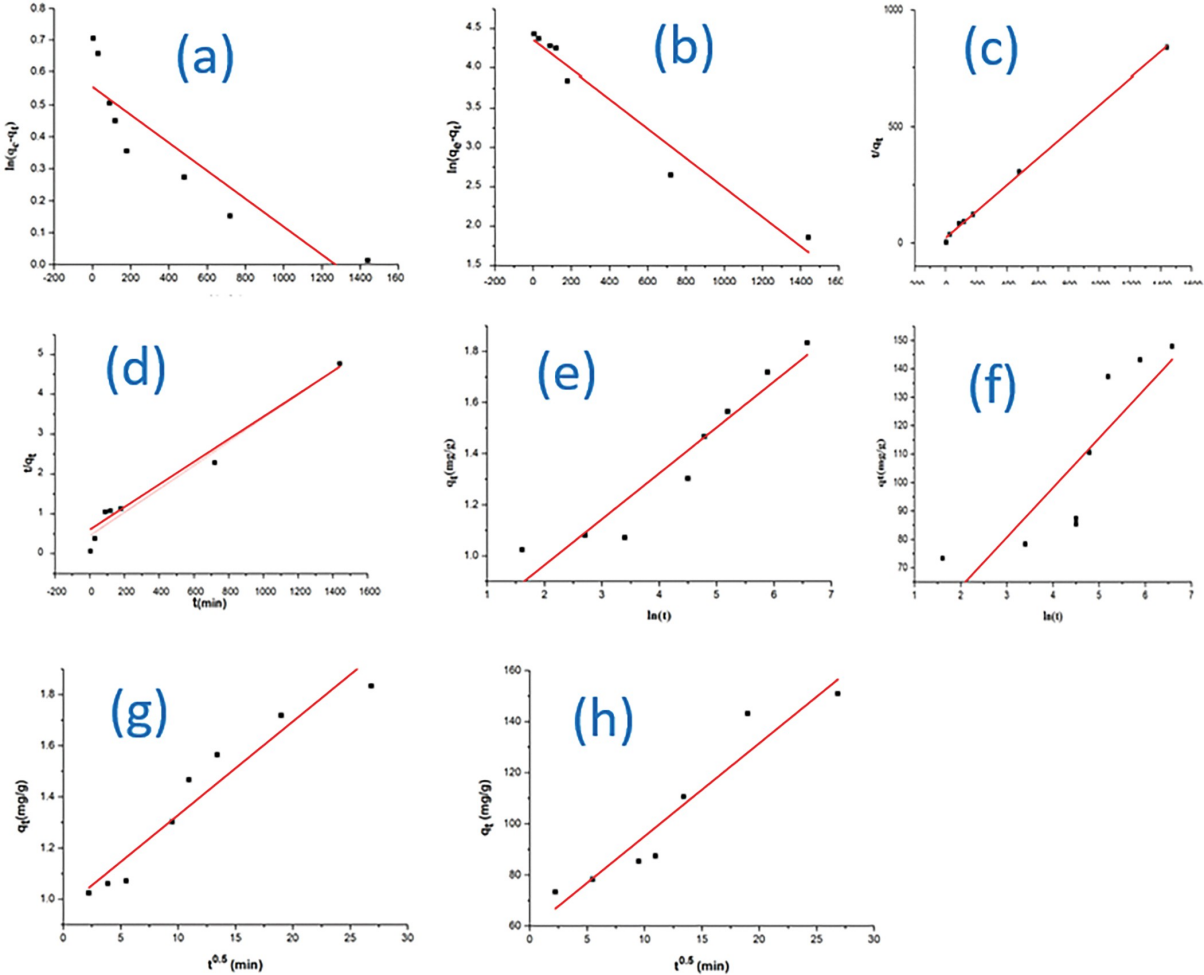
Pseudo-first-order kinetics model for the adsorption **(a)** Cd(II); **(b)** Pb(II) ions; Pseudo-second-order kinetics model for the adsorption **(c)** Cd(II) **(d)** Pb(II) ions; Elovich model for the adsorption **(e)** Cd(II) **(f)** Pb(II) ions, (g) intraparticle diffusion model for the adsorption of Cd(II) (h) Pb(II) ions on oyster shell biocomposite.

The kinetic modelling was carried out on the experimental data to investigate the rate of metal ions adsorbed on the oyster shell biocomposite. The results of the pseudo-first- order, pseudo-second-order, Elovich and Intraparticle diffusion parameters are given in **Table 8** and represented in **Fig 9A–9h.**

**Table 8:** Adsorption kinetic constants of Pseudo-first-order, Pseudo-second order, Elovich and Intraparticle diffusion models on Oyster shell biocomposite.

Metal	Pseudo- first- order		Pseudo- second- order		Elovich			Intraparticle	
	K_1_(min^-1^)	R^2^	k_2_ (g/(mg min))	R^2^	α (mg g^−1^ min^−1^)	β (g mg^−1^)	R^2^	*k*_*i*_ (mg g−1 min−1/2)	R^2^
Cd(II)	4.379x10^-5^ ±0.051	0.7839	750.54±5.74	0.9982	0.17951±0.01	0.6047±0.00042	0.9033	0.9625±0.0004	0.9156
Pb(I)	1.87x10^-2^ ±0. 57	0.9549	69.41±0.15	0.9621	1.74813±0.0006	2.8263±0.0003	0.7012	3.6428±0.0035	0.8988

It was revealed that the values of regression coefficient of kinetic data fitted most to the pseudo-second order model for both metals under study. Furthermore, theoretical q_e_ values were closer to the experimental values for pseudo-second-order kinetics implying that the pseudo-second-order model best described the adsorption of Cd(II) and Pb(II) on the adsorbent. The intra-particle diffusion kinetic model based on the theory or equation proposed by Weber and Morris was used to study the diffusion mechanism. The kinetic data fitted to the intraparticle model (**Fig 9G and 9H**) indicated a linear fit of intra-particle diffusion of the model for adsorption of heavy metal ions onto the oyster-silver biocomposite adsorbent. Generally, the result revealed that the plots present multilinearity which implies that three steps described the diffusion of metals on the sites of the adsorption [52]. Firstly, sharper region indicates the instantaneous adsorption which was achieved within the first 5 min. indicating that at the initial stage of adsorption the diffusion of increased initial heavy metal ion concentration was the predominant feature. The second stage signified the gradual adsorption stage where intra-particle diffusion is the rate limiting step. The third stage which is the final diffusion stage indicate the final equilibrium stage whereby the intra-particle diffusion further slows down due to the low concentrations of heavy metal ions left in the solutions. It was revealed that the initial state favoured higher diffusion of Pb(II) on the adsorbent sites. This could be attributed to the chemical affinity of the CaCO_3_ enriched biocomposite and the derivative solubility products of PbCO_3_ [53].

The efficiency of the oyster she**ll** biocomposite for the adsorption of Cd(II) and Pb(II) ions was compared with other adsorbents in the literature based on the maximum adsorption capacity as indicated in **Table 9.** The result signified the suitability of the biocomposite as effective adsorbent. Also, its prospect as alternative adsorbent for the biosorption of metal ions from solution was enhanced by its higher maximum adsorption capacity.

**Table 9:** Adsorption capacity of adsorbents for the removal of Cd(II) and Pb(II) ions from solution.

Adsorbate	Adsorbent	Q _max_ (mg/g)	Ref
Cd(II) ions	Untreated fir cone powder	3.74	[53]
Cd(II) ions	Fly ash	1.22	[54]
Cd(II) ions	Banana peel	5.71	[33]
Cd(II) ions	Fly ash-chitosan composite	87.72	[55]
Cd(II) ions	Graphene oxide membrane	83.8	[56]
Cd(II) ions	Alumina-decorated multi-walled carbon nanotubes	27.21	[57]
Cd(II) ions	Maize tassel-magnetite nanohybrid adsorbent	52.05	[58]
Cd(II) ions	Oystershell biocomposite	97.54	This study
Pb(II) ions	Silica nanopowders/alginate composite	36.51	[59]
Pb(II) ions	Attapulgite/chitosan (ATP/CS) composite	67.8	[60]
Pb(II) ions	Chitin-Halloysite nanoclay	8.2	[61]
Pb(II) ions	Graphene oxide-polyvinyl alcohol coated sand composite Oystershell biocomposite	16.56	[62]
Pb(II) ions	Thiol-functionalized cellulose nanofiber membrane	22	[63]
Pb(II) ions	Chitosan-palygorskite (MCP) nanocomposite	58.5	[64]
Pb(II) ions	chitosan/B. longum composite	3.93	[65]
Pb(II) ions	Oystershell biocomposite	78.989	This study

#### 3.5.3 Thermodynamic studies

The adsorption of Cd and on the modified oystershell biocomposite was carried out at 303-328K to investigate the thermodynamic parameters using Van’t Hoff equation. The result is presented in **Table 10**. The enthalpy ΔH for Cd and Pb removal were 10.348 **and 16.13 *kJ/mol*** respectively suggesting that the process of adsorption was endothermic in nature. The enthropy **Δ*S* at** 303K was positive (46.852 and 68.207 J/mol K) for Cd and Pb respectively which signifies that high degree of randomness and strong afinity of ions occurred for the modified oyster shell biocomposite. The Gibbs free energy which is a measure of the spontaneity of a reaction process was found to increase negatively as the temperature increased for both Cd and Pb adsoprtion which indicated favorbale and spontaneous adsoprtion on the modified oyster shell biocomposite.

**Table 10:** Thermodyanmic parameters of Cd and Pb metals adsorption.

	Δ*G* (kJ/ mol)				Δ*H*	Δ*S*
	303K	318K	323K	328K	*(kJ/mol)*	(J/mol K)
Cd(II) ions	-3.504	-3.828	-4.004	-4.258	10.348	46.852
Pb(II) ions	-4.641	-5.251	-5.943	-6.405	16.130	68.207

### 3.6 Regeneration and reuse

**Fig 10:**
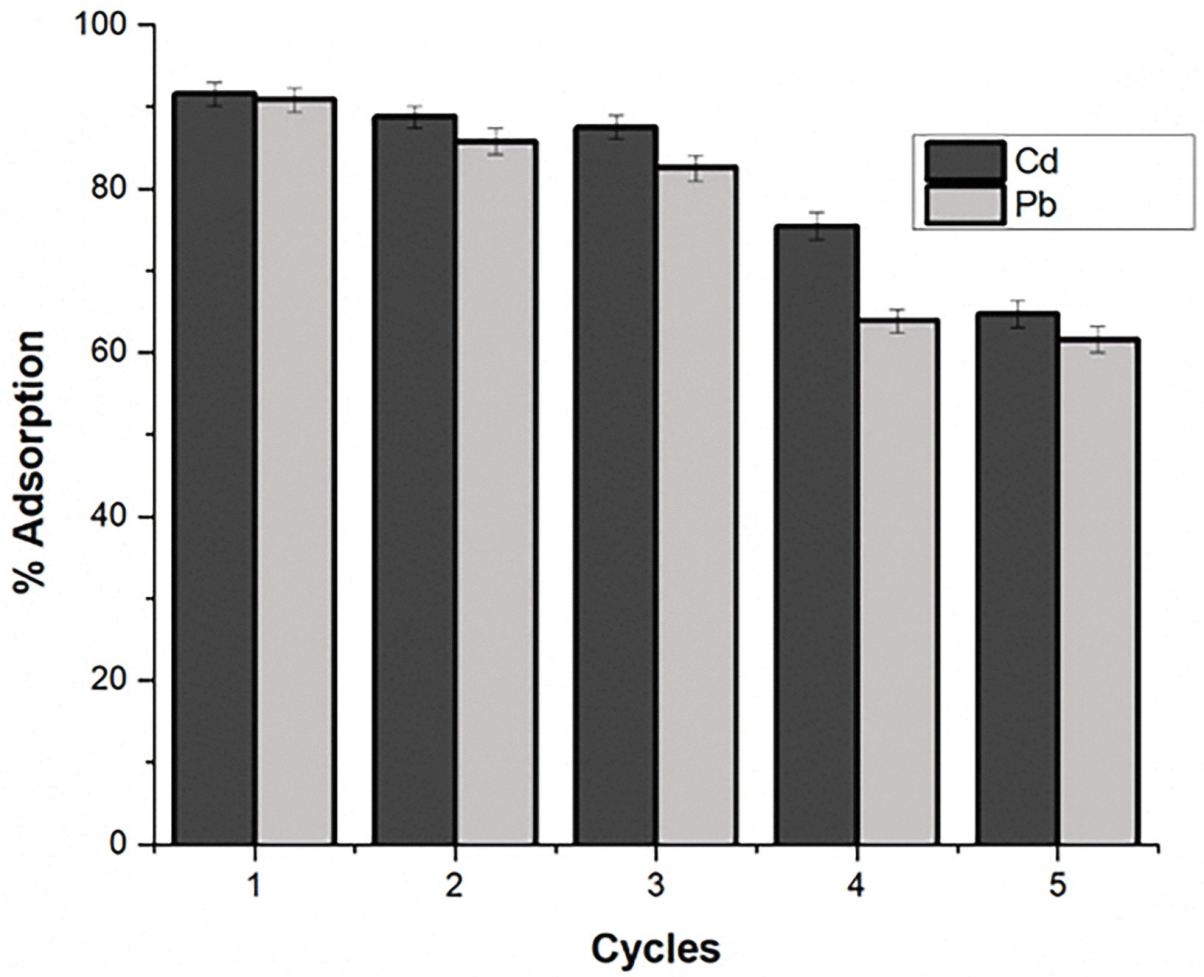
Reusability of oyster shell biocomposite in continuous batch adsorption (five cycles).

Regeneration and reuse assays were conducted with five consecutive adsorption/desorption cycles on the spent oystershell biocomposite **(Fig 10).** The removal of Cd decreased with increasing cycle without significant difference in the adsorption capacity in the first two cycles. There was a slight decrease in the third and fourth cycle until a steep decline of the removal efficiency of the biocomposite was achieved in the fifth cycle the precent removal of Pb was noticed to decrease after the first cycle. The next two cycles achieved decline in the adsorption capacity after which there was stability (%) in the removal after the third cycle denoting the reusability of the oystershell biocomposite retaining its adsorption capacity for Cd and Pb removal from aqueous solution which suggests its prospect for watewater applications.

### 3.7 Point zero charge study of oyster shell biocomposite adsorbent

**Fig 11:**
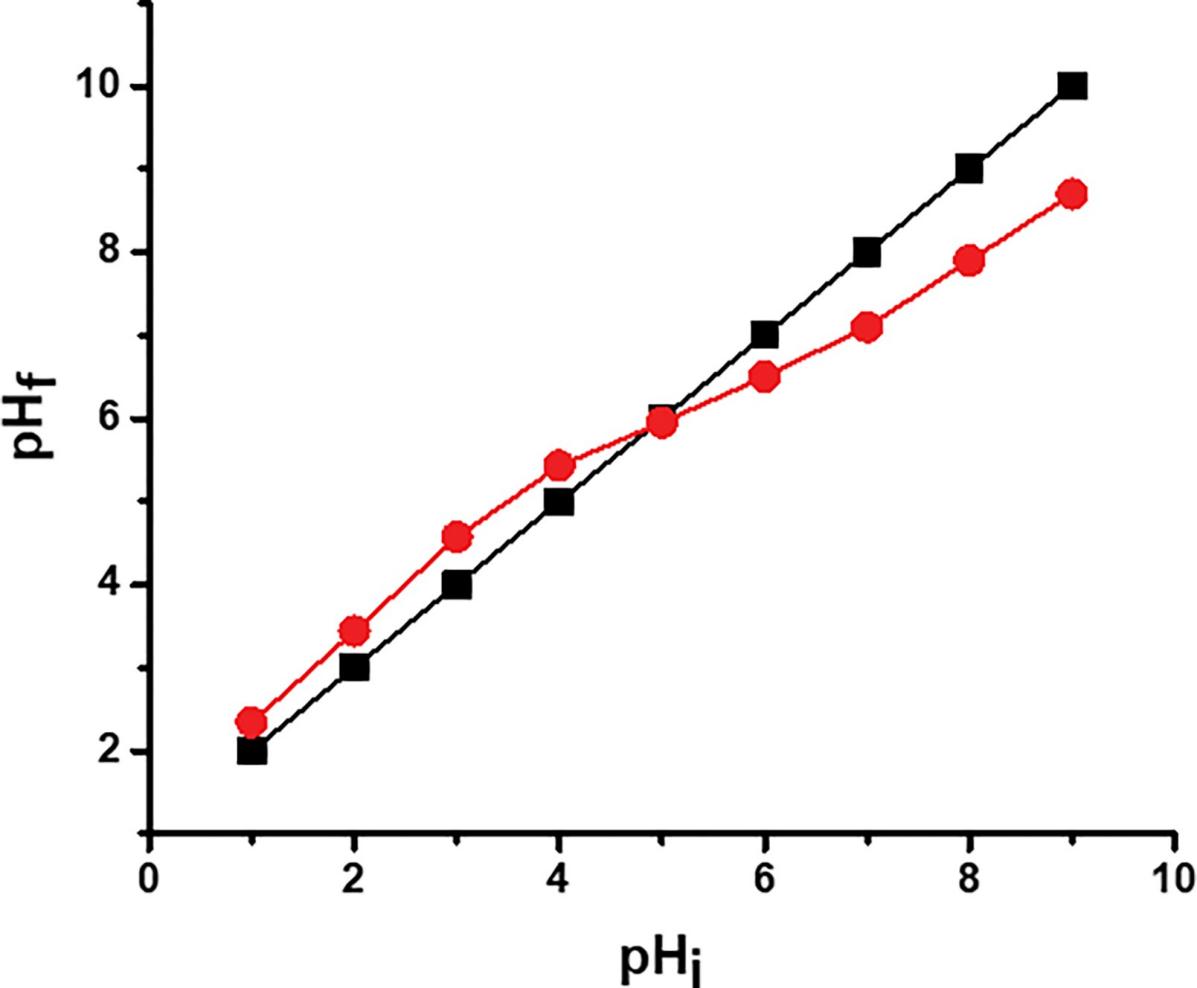
Point zero charge study of oyster shell biocomposite adsorbent.

The biosorption of Cd(II) and Pb(II) ions onto oyster shell biocomposite adsorbent was influenced by the pH of the solution. Cd(II) and Pb(II) absorption was found to increase as the pH of the solution increased with maximum adsorption achieved at pH 5.57, then declined as the pH of the solution increases (**Fig 11**). Oyster shell biocomposite particles’ pH_ZPC_ was found to be 5.1. The pH_PZC_ indicated no net charge present on the adsorbent surface. Below the pH_PZC_, the excess of H^+^ present on the surface of oyster shell biocomposite repelled the positively charged cationic Cd(II) and Pb(II). However, when the mixture’s pH was greater than pH_ZPC_, the net surface charge was negative due to the desorption of H^+^, and Cd(II) and Pb(II) uptake due to coulombic interaction. The mesoporous oyster shell biocomposite pHpzc was close to 5.1, indicating that the adsorbent surface had a negative charge and was more amenable to the adsorption of positively charged Cd(II) and Pb(II) ions at pH levels higher than pHpzc. As a result, raising pH considerably improved Cd(II) and Pb(II) ability to adsorb. At higher pH, the precipitation occurred due to formation of metal hydroxide.

### 3.8. Possible biosorption mechanism

**Fig 12:**
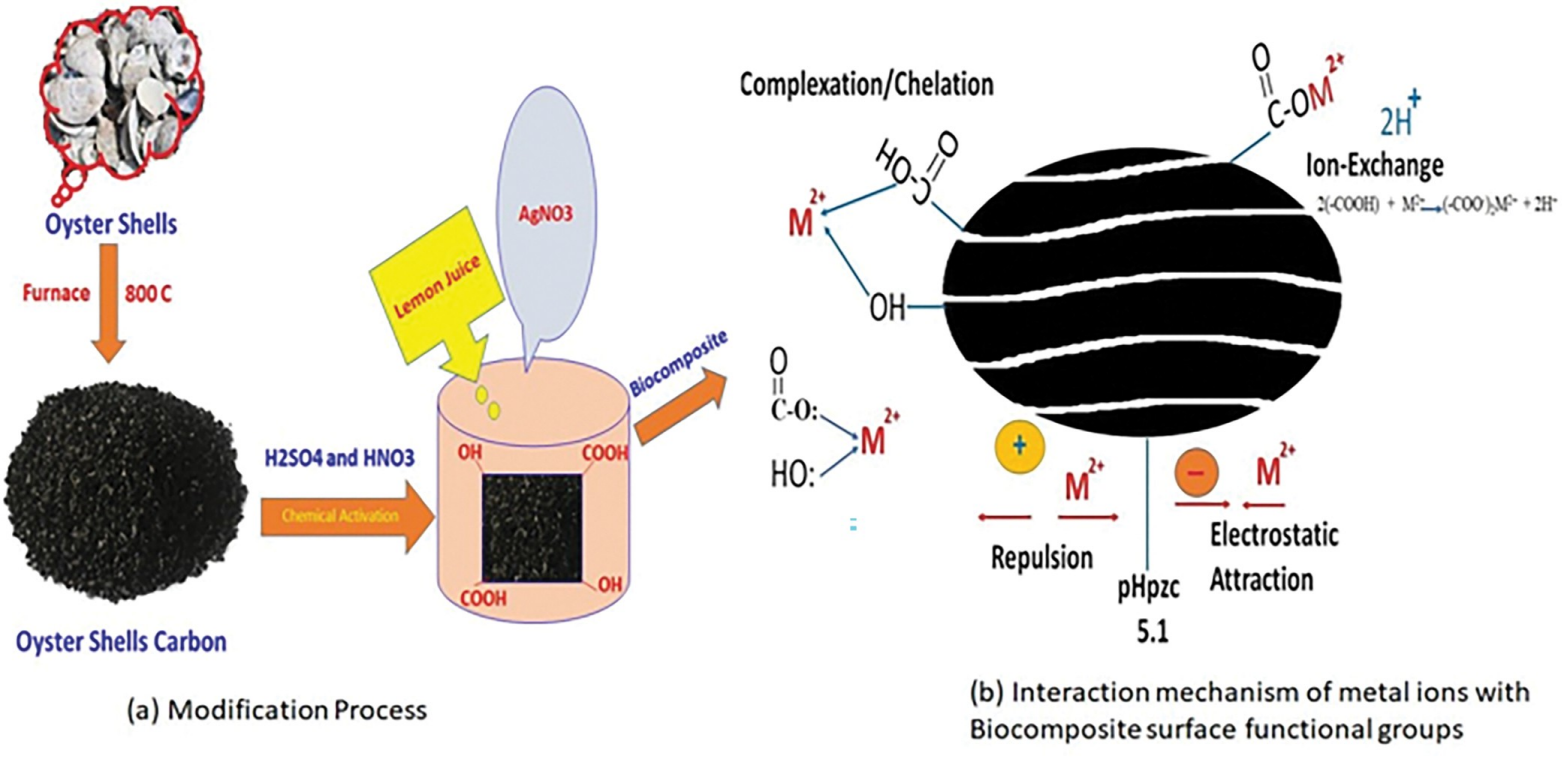
**(a)**Modification road map of oyster shell biocomposite **and (b):** Interaction and binding mechanism of Cd(II) and Pb(II) metals with oyster shell biocomposite surface functional group.

Modification road map of oyster shell biocomposite was represented in **Fig 12a**. The analysis of the FTIR spectra confirmed that carbonyl, hydroxyl and carboxylic group were the predominant functional groups on the surface of the oyster shell biocomposite adsorbent. The sorption of metal ions onto the surface of oyster shell biocomposite may be owing to the chelation, electrostatic interaction and ion-exchange process as indicated by the EDX spectra before and after adsorption. Due to the chemical activation (with sulphuric and nitric acid) of oyster shell carbon, mainly carboxylic and hydroxyl groups were fixed with the surface of the oyster shell biocomposite. The increase in adsorption as a result of temperature increase possibly could be attributed to intra particle diffusion within the pores due to change in pore sizes. As a result of the modification of oyster shell biocomposite relative to the oyster shell powder, the BET surface area of the biocomposite was found to increase therby increasing the adsorption capacity on a monolayer surface. This facilitates increase in chemical affinity of Cd(II) and Pb(II) ions and its binding effect on the adsorbent resulting to increase in adsorption capacity. Since diffusion is an endothermic process, increased adsorption is most likely at higher temperature due to increase in diffusion rate of ions in the external mass transport. These functional groups have oxygen atom which has lone pair of electrons. Some of following factors are responsible for bonding the metal ions with oyster shell composites Fig 12A and 12B.

- Complexation/ Chelation: These groups (Carboxylic and hydroxyl) have pair of electrons on oxygen atoms and can easily donate their lone pair electron to metal ions and make a co-ordination bond between carboxylic, hydroxyl and metal ions.
  2(-COOH) + M^2+^………….. (-COO^-^)_2_M^2+^ + 2H^+^
  Here, (-COOH) and M^2+^ represents the functional group presents on the surface of biocomposite and metal ions (Cd and Pb).
- Ion-exchange process: Hydrogen ions of carboxylic group can be replaced with metal ions. With this process, metal ions may be removed from the aqueous solution using oyster shell biocomposite adsorbent.
- Electrostatic attraction/Coulombic attaction: Cd(II) and Pb(II) ions adsorption onto oyster shell biocomposite adsorbent was influenced by the pH of the solution. Cd(II) and Pb(II) absorption increased as the pH of the solution increased and maximum at pH 5.57, then declined as the pH of the solution increases. In terms of the pH point zero charger (pH_pzc_), oyster shell biocomposite particles’ pH_PZC_ was found to be 5.1 as discussed in section 3.7.

Below the pH_PZC_, the excess of H+ present on the surface of oyster shell biocomposite repelled the positively charged cationic Cd(II) and Pb(II). However, when the mixture’s pH was greater than pH_P_z_C_, the net surface charge appeard negative due to to coulombic interaction resulting to the desorption of H^+^, and Cd(II) and Pb(II) uptake. The mesoporous oyster shell biocomposite pHpzc was close to 5.1, indicating that the adsorbent surface had a negative charge and was more amenable to the adsorption of positively charged Cd(II) and Pb(II) ions at pH levels higher than pHpzc. As a result, raising pH considerably improved Cd(II) and Pb(II) ability to adsorb. At higher pH, the precipitation occurred due to formation of metal hydroxide (**Fig 12A**).

Hence, from this process, it can be affirmed that the modified oyster shell biocomposite exhibited strong ion exchange capacity and effective binding potential of metal ions on its surface. The mechanism of adsorption of Cd(II) and Pb(II) ions on oyster shell biocomposite is illustrated in **Fig 12B**.

## 4. Conclusions

The adsorption study of Cd and Pb removal revealed that the oyster shell biocomposite exhibited an improved surface area from the result of the BET specific surface area which indicated that mesoporous surface of the biocomposite structure influenced the adsorption of Cd and Pb. The FT-IR study indicated that the binding of both metal ions on the surface of the oyster shell biocomposite was influenced by the presence of carbonyl, carboxyl and hydroxyl functional groups. This asymmetric vibration of Raman spectra around 1560–1599.77 cm^-1^ revealed that the interaction between the carboxylate groups on the oyster shell composite and the Ag (II) ions resulting in the effective adsorption of Cd (II) and Pb(II) ions. A 2^4^ full factorial central composite design for the batch adsorption was implemented using (Design Expert 6.0.4) software for the prediction of the result of 30 experimental runs conducted for the adsorption of Cd(II) and Pb(II) ions onto oyster shell biocomposite. The relationship between the experimental and predicted data was found to achieved high correlation (R^2^ > 92%) for both metal ions. The uptake of Cd(II) revealed to be more sensitive to the effect of the interaction of process variables of pH, adsorbent dosage, initial concentration and temperature. Meanwhile, the adsorption of Pb(II) ions was found to be more sensitive to the linear effect of adsorbent dosage, initial concentration and temperature. In general, effective adsorption of both metal ions was optimally achieved at pH 5.57, adsorbent dosage of 2.53 g/L, initial concentration of 46.76 mg/L at 24.48°C. The response surface methodology proved to be an effective tool for the process optimization. The biosorption of both metal ions followed the Langmuir isotherm suggesting monolayer adsorption. The kinetic data of biosorption of Cd(II) and Pb(II) ions fitted better to the pseudo-second order model. It was revealed that the biosorption of both metal ions was spontaneous and rapid process. The FT-IR study indicated that the binding of both metal ions on the surface of the oyster shell biocomposite was influenced by the presence of carbonyl, carboxyl and hydroxyl functional groups. The study has found oyster shell biocomposite as a potential biosorbent for the uptake of metal ions from aqueous solution.
